# Retrospective and multifactorial single-cell profiling reveals sequential chromatin reorganization during X inactivation

**DOI:** 10.1038/s41556-025-01687-w

**Published:** 2025-07-10

**Authors:** Samy Kefalopoulou, Pim M. J. Rullens, Kim L. de Luca, Sandra S. de Vries, Tessy Korthout, Alexander van Oudenaarden, Peter Zeller, Jop Kind

**Affiliations:** 1 Hubrecht Institute, Royal Netherlands Academy of Arts and Sciences (KNAW) and University Medical Center Utrecht, Utrecht, The Netherlands; 2 Oncode Institute, Utrecht, The Netherlands; 3 Department of Molecular Biology, Faculty of Science, Radboud Institute for Molecular Life Sciences, Radboud University Nijmegen, Nijmegen, The Netherlands; 4 Present Address: Department of Molecular Biology and Genetics, Aarhus University, Aarhus, Denmark

**Keywords:** Histone analysis, Next-generation sequencing, Nuclear envelope, Epigenomics, Dosage compensation

## Abstract

The regulation of gene expression is governed at multiple levels of chromatin organization. However, how gene regulation is co-ordinated remains relatively unexplored. Here we develop Dam&ChIC, a method that enables retrospective and multifactorial chromatin profiling in single cells. Dam&ChIC employs chromatin labelling in living cells with ^m6^A to acquire a past chromatin state, coupled with an antibody-mediated readout to capture the present chromatin state. Analyses of diverse factor combinations highlight its versatility and superior resolution. By tracking lamina-associated domain inheritance over the cell cycle, we showcase that Dam&ChIC provides retrospective single-cell chromatin data. When applied in random X chromosome inactivation, Dam&ChIC disentangles the temporal order of chromatin remodelling events. Upon mitotic exit and following Xist expression, the inactive X chromosome undergoes extensive genome–lamina detachment, preceding spreading of Polycomb. We anticipate that Dam&ChIC will be instrumental in unravelling the interconnectivity and order of gene-regulatory events underlying cell-state changes during development.

## Abstract (web summary)

Kefalopoulou, Rullens et al. develop Dam&ChIC to assay chromatin state at two different time points in the same cell. The method was used to study the reorganization of LADs during cell division and X chromosome inactivation.

## Main

Gene regulation is orchestrated by multiple factors at different scales of chromatin organization. Among others, post-translational modification of histone tails (hPTMs)^1^, the higher-order folding of chromatin^2^ and genomic interactions of chromatin with nuclear scaffolds, such as the nucleolus and the nuclear lamina (NL)^3^, play key roles in regulating gene activities.

Over the past decade, progress in molecular technologies enabled profiling many such features of chromatin organization in single cells^4–18^, revealing more extensive cell-to-cell variability and dynamics than anticipated. To assess the interconnectivity between chromatin features, recent methods enabled simultaneous measurements of multiple factors from the same cell. Approaches such as multi-CUT&Tag^19^, MulTI-Tag^20^, nano-CT^21^, NTT-seq^22^ and scMAbID^23^ provide multifactorial information by using combinations of target-specific antibodies and in situ tagging of the chromatin. Despite their applicability and potential to reveal gene-regulatory hierarchies, the sparsity of information obtained by some of these methods poses a major challenge. As a result, our understanding of multifactorial control of gene regulation remains limited.

Furthermore, in situ tagging methods capture static end points of chromatin states and lack dynamic capabilities. Sequencing tools for recording chromatin state transitions over time in the same cell remain an unresolved challenge; yet one that could provide unique insights into the direct role of chromatin dynamics in determining cellular outcomes. The capacity for molecular recording in eukaryotes is inherent to a few existing systems, based on expression of bacterial methyltransferases fused to chromatin factors^24–28^. These fusion enzymes can label the genome with methylation marks in proximity to the sites of interaction with the chromatin. As their metabolic pathways are absent in eukaryotic cells, such labels can be retained over time and thus represent molecular footprints of past protein–DNA interactions that can be retrieved upon sequencing.

A single-cell technology with the ability to record protein–DNA interactions over a controlled period of time in vivo is (sc)DamID^7,24–27,29^. DamID involves the fusion of a protein of interest (POI) to the *Escherichia* *coli* methyltransferase Dam that, upon conditional expression in live cells, deposits adenine-6-methylation (^m6^A). This mark is cumulative and remains stable until DNA replication, thereby reflecting the genomic occupancy of the POI over time. However, information by scDamID alone is not sufficient to disentangle past and present chromatin states within the same cell.

To obtain both past- and present-state information, we hypothesized that scDamID could be coupled with an approach that provides snapshot readouts at the time of cell collection. An ideal candidate technology for this purpose is sortChIC^30^, which recovers high-resolution single-cell chromatin profiles through in situ targeting of micrococcal nuclease (MNase) fused to protein A (pA-MNase), to specifically digest and amplify antibody-bound chromatin^31,32^. Building upon these two approaches, here we developed Dam&ChIC, a single-cell technology that combines recording of chromatin states in living cells and antibody-directed chromatin digestion in situ, with two capabilities: (1) multifactorial measurements at high resolution, to dissect the interplay between chromatin states in single cells; and (2) retrospective measurements within the same cell, to uncover chromatin dynamics over time.

Here, we demonstrate that Dam&ChIC recovers high-quality multifactorial chromatin data, benchmarked against state-of-the-art methods. We provide the proof-of-concept for single-cell retrospective measurements, by studying the reorganization of lamina-associated domains (LADs) over the cell cycle. We employ Dam&ChIC in X-chromosome inactivation (XCI) and, unexpectedly, we uncover extensive detachment of the inactive X from the lamina that occurs after expression of the XCI master regulator Xist, but precedes accumulation of Polycomb hPTMs. Initiation of this chromosome-wide detachment coincides with mitotic exit. Collectively, Dam&ChIC provides an experimental and analytical framework to study hierarchies of chromatin regulatory events associated with cell-state transitions in single cells.

## Results

### Method design

The development of Dam&ChIC involves integration of two methodologies that are fundamentally different. In particular, DamID requires creating a cell line that conditionally expresses a Dam–POI to deposit ^m6^A on proximal genomic GATC motifs over a desired time window. sortChIC involves antibody-directed pA-MNase activity to capture a static end-state in nuclei or fixed cells.

**Fig. 1:**
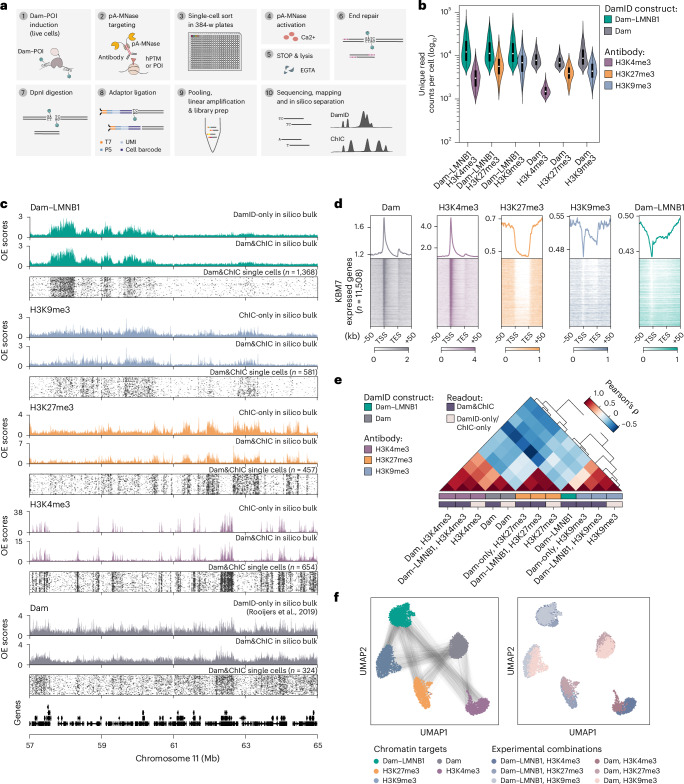
Dam&ChIC design and mapping of diverse chromatin feature combinations in single cells. **a**, Graphical overview of the single-cell Dam&ChIC method. **b**, Violin plots depicting the unique number of reads obtained per cell by Dam&ChIC for different combinations of Dam constructs and antibodies. Violins show a kernel density estimation of the distribution of data points. **c**, Single-cell heatmaps of each chromatin feature profiled with Dam&ChIC. In silico bulk profiles are shown as OE values. Corresponding in silico bulk profiles of ChIC-only datasets in KBM7 cells are shown for comparison. For the untethered Dam, the in silico bulk of a corresponding publicly available DamID-only dataset in KBM7 cells^20^ is used for comparison. **d**, Heatmaps showing chromatin features profiled with Dam&ChIC aligned on KBM7 expressed genes. Genes (rows) are ordered on their relative expression levels, determined by publicly available RNA-seq data^29^. Line plots indicate scaled averages of the heatmaps for each mapped chromatin feature. **e**, Hierarchical clustering depicting genome-wide Pearson correlations relating all in silico bulk Dam&ChIC chromatin data and corresponding ChIC-only datasets, or a publicly available DamID-only dataset^29^. Data were binned in 1-kb bins. Data labelled as ‘Dam’ or ‘Dam–LMNB1’ (Dam&ChIC colour label), contain the respective DamID readouts from all experimental combinations with hPTMs. **f**, UMAP representations of Dam&ChIC data binned in 100-kb genomic bins. Each cell (dot) is represented twice, once for each measurement. Left; coloured by chromatin target, right; coloured by experimental combination. Black lines in the left UMAP connect the same cell. Source numerical data are available in source data. Source data

**Extended Data Fig. 1:**
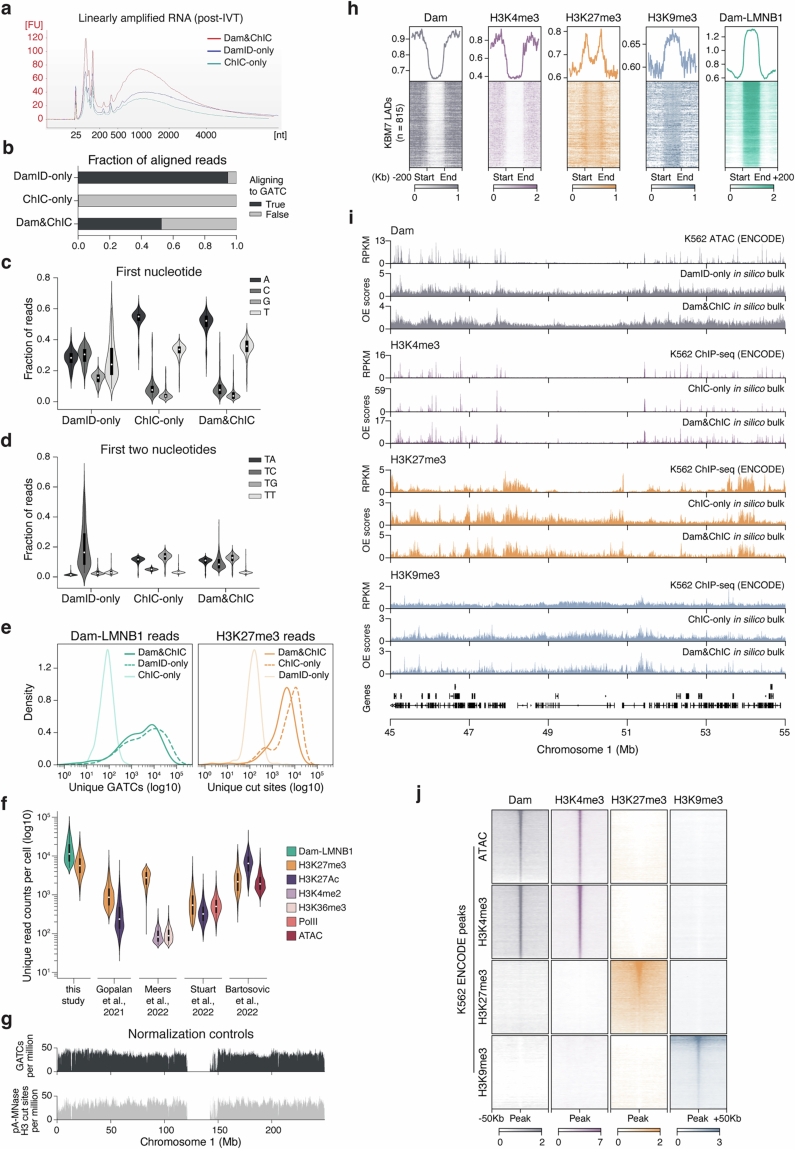
Benchmarking of Dam&ChIC data against state-of-the-art methods. (**a**) Bioanalyzer plot showing the amount and size distribution of linearly-amplified RNA (aRNA), derived after *in vitro* transcription (see Methods). Dam&ChIC was performed to profile Dam-LMNB1 and H3K27me3, alongside respective control DamID-only and ChIC-only experiments. Each aRNA product was derived from a 384-well plate containing 380 cells, barcoded and pooled together prior to amplification. The samples were processed in parallel. (**b**) Comparison of the fraction of reads mapping on a genomic GATC motif (black) between Dam&ChIC, DamID-only and ChIC-only. (**c**) Violin plots showing a quantification of the first nucleotide of invalid DamID reads (that is, not mapping on genomic GATC motifs) for Dam&ChIC, DamID-only and ChIC-only. (**d**) Violin plot showing a quantification of the first two nucleotides of invalid DamID reads for Dam&ChIC, DamID-only and ChIC-only. (**e**) Distribution of the unique number of Dam-LMNB1 (left) or H3K27me3 (right) reads recovered per cell by DamID-only, ChIC-only or Dam&ChIC. (**f**) Comparison of unique number of reads obtained per cell between Dam&ChIC and other multifactorial chromatin profiling methods^19–22^. (**g**) Genomic distribution of *in silico* GATC mappability (top, black) and mappability of pA-MNase cut sites derived experimentally with ChIC against H3 (bottom, grey). (**h**) Heatmaps showing chromatin features profiled with Dam&ChIC, aligned over LADs (n = 815) that were previously defined in KBM7 cells^29^. Line plots indicate scaled averages of the heatmaps for each mapped chromatin feature. (**i**) Genomic tracks of *in silico* bulk Dam&ChIC profiles compared to corresponding ChIC-only datasets in KBM7 cells and corresponding ENCODE (ENCODE 2012) ChIP-seq or ATAC-seq datasets in K562 cells. (**j**) Tornado plots depicting *in silico* bulk Dam&ChIC data aligned on ENCODE ChIP-seq or ATAC-seq peak coordinates in K562 cells. Peaks (rows) are ordered on the peak signal of the corresponding Dam&ChIC dataset (that is, ATAC-seq peaks are ordered on Dam DamID data, H3K4me3 peaks are ordered on H3K4me3 ChIC data and so on). Source numerical data is available in source data. Source data

In Dam&ChIC, Dam expression is induced in living cells (Fig. 1a, step 1), which are subsequently collected, permeabilized or fixed, and stained with an antibody of interest (Fig. 1a, step 2). Afterwards, single nuclei are sorted in 384-well plates via fluorescence-activated cell sorting (FACS) (Fig. 1a, step 3) and further processed with robotic liquid handling (Methods). The molecular processing includes activation of pA-MNase (Fig. 1a, steps 4–5), blunt-ending of the pA-MNase-produced fragments (Fig. 1a, step 6), DpnI digestion to specifically enrich for genomic fragments containing ^m6^A-marked GATC motifs (Fig. 1a, step 7), and ligation of both types of fragments with blunt-end forked adaptors (Fig. 1a, step 8). The adaptor design includes a T7 promoter for in vitro transcription (IVT), unique molecular identifiers (UMIs), the Illumina P5 sequence and cell-specific barcodes^29,33^, allowing linear amplification of the produced fragments and Illumina library preparation (Fig. 1a, step 9 and Extended Data Fig. 1a). Upon high-throughput sequencing, the unique sequence context of DamID and ChIC reads is leveraged to separate the pool of fragments in silico (Fig. 1a, step 10 and Methods). DamID reads almost exclusively align to genomic GATC motifs, contrary to ChIC-derived reads that do not display motif specificity (Extended Data Fig. 1b). pA-MNase has intrinsic preference for A/T-rich genomic regions^34^, and indeed, ~95% of ChIC reads start with either an A or T nucleotide (Extended Data Fig. 1c,d), which we use to achieve more confident read separation (Methods). Notably, the number of reads recovered for both modalities, is similar to when either method is performed individually (control datasets are henceforth referred to as DamID-only and ChIC-only) (Extended Data Fig. 1e). In the DamID-only and ChIC-only libraries, on-target reads separate from off-target signal by at least two orders of magnitude (Extended Data Fig. 1e). Therefore, the Dam&ChIC protocol yields sequencing libraries containing both DamID- and ChIC-derived fragments, which can be separated in silico based on sequence context.

### Dam&ChIC maps diverse chromatin types with high resolution

To benchmark the integrated protocol and test its versatility in profiling different chromatin features, we generated single-cell data of diverse combinations of heterochromatic and euchromatic chromatin types. We used two previously established human KBM7 cell lines that conditionally express either Dam fused to the core lamina protein LMNB1 or untethered Dam. Dam–LMNB1 has been previously used to characterize LADs in single cells^7^, while the untethered Dam enzyme marks accessible chromatin in single cells^29^. KBM7 cells have a near-complete haploid genome, ensuring that both Dam&ChIC measurements originate from the same chromosome copy. Expression of Dam–LMNB1 or untethered Dam in live cells was induced for 15 h, to enable ^m6^A deposition. Thereafter, we stained nuclei with antibodies specific to the hPTMs H3K4me3, H3K27me3 or H3K9me3. Additionally, we performed ChIC-only experiments for the same set of hPTMs and used matching DamID-only data, both derived from KBM7 cells.

For Dam–LMMB1 and the untethered Dam, Dam&ChIC recovers a median of ~12,300 and ~8,500 UMI-flattened (unique) reads per cell respectively, in all combinations (Fig. 1b). For the euchromatic H3K4me3 it recovers a median of ~2,600 unique reads per cell and for the heterochromatic H3K27me3 and H3K9me3 modifications a median of ~5,000 and ~5,500 unique reads per cell, respectively (Fig. 1b). Dam&ChIC thereby attains higher sensitivity compared with recently published multifactorial chromatin profiling methods, particularly considering the ploidy of KBM7 cells (Extended Data Fig. 1f). To normalize the two fragment types in the Dam&ChIC libraries, we computed observed over expected (OE) scores^7^. For the DamID readout, OE scores are calculated over the in silico genomic distribution of GATC motifs (Extended Data Fig. 1g and Methods). For ChIC, the distribution of maximum expected reads was generated with a bulk ChIC experiment against H3 (Extended Data Fig. 1g and Methods).

Further benchmarking of Dam&ChIC to ChIC-only and DamID-only data or matching ENCODE data from K562 cells, confirms highly specific single-cell genomic enrichment at the expected regions (Fig. 1c,d and Extended Data Fig. 1h–j). Moreover, correlation between Dam&ChIC and the corresponding ChIC-only and DamID-only datasets is high (Fig. 1e). Finally, dimensionality reduction of the entire Dam&ChIC dataset reveals consistent separation of cells based on chromatin type (Fig. 1f, left), regardless of the used experimental combinations (Fig. 1f, right). Altogether, these results demonstrate that Dam&ChIC is a versatile multifactorial method for profiling euchromatic and heterochromatic factors in single cells with high sensitivity and specificity.

### Untangling past and present chromatin states in single cells

**Fig. 2:**
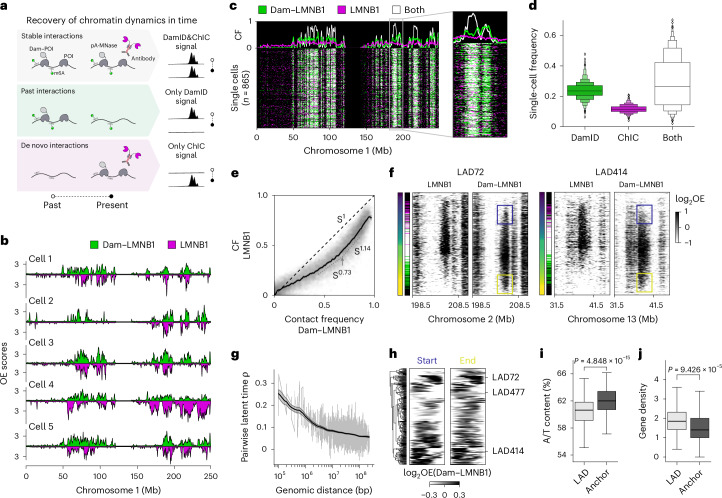
Single-cell retrospective profiling of genome–lamina interactions. **a**, Schematic of the chromatin dynamics that Dam&ChIC resolves between the time of Dam–POI induction (past) and collection (present). **b**, ‘Mirror plots’ of five example cells showing Dam&ChIC LAD signal, as OE values in 1,100-kb-bins. **c**, Combined single-cell heatmap with binarized LAD signal in 100-kb genomic bins, detected by either DamID (green), ChIC (magenta) or both measurements (white). Line plots represent the CF, which is the fraction of cells for which a bin is detected by DamID, ChIC or both. **d**, Quantification of the frequency by which each LAD is measured in a single cell by DamID, ChIC or both. Boxen plot shows a large number of quantiles to provide details of data distribution. **e**, Scatter-plot comparing the CF of DamID and ChIC measurements of Dam&ChIC. Black uninterrupted line indicates rolling mean, with 95% confidence interval. Diagonal dashed line indicates complete agreement between both measurements. Slope is quantified at different positions. **f**, Single-cell heatmaps for DamID and ChIC for two LADs, with cells ordered by latent time (left-most gradient bar). Middle bar indicates LAD state, with the same colours as used in **c**. LAD signal is log-transformed OE in 100-kb genomic bins. Blue and yellow boxes indicate establishment and release of each domain, from and towards the same genomic location. **g**, Pairwise Pearson’s correlation coefficient of latent time assignment between any pair of LADs, plotted against genomic distance. Black line indicates rolling mean, with 95% confidence interval. **h**, Hierarchical clustering of latent time-averaged start and end positions of LADs (‘anchors’ where LADs start attaching or releasing). Latent time averages are from 50 cells up and downstream of start or end, as depicted in blue and yellow boxes, respectively in **f**. LAD signal is log-transformed OE in 100-kb bins. **i**,**j**, Quantifications of A/T content (**i**) and gene density (**j**) in 100-kb genomic bins on anchor regions (Methods) compared with the rest of the LAD. Box shows the quartiles of data and whiskers extend to the full distribution. Two-sided *t*-test *P* value = 4.848 × 10^−15^ (**i**) and *P* = 9.426 × 10^−5^ (**j**). Source numerical data are available in source data. Source data

Previous findings with a DamID-microscopy technology indicated that over a 15-h time window, LAD dynamics are extensive, yet constrained to a 1-µm zone underneath the NL, and they can be efficiently detected by DamID^35^. In contrast to DamID, ChIC detects LADs that are in contact with the lamina at the time of cell collection. To explore the possibility to capture temporal dynamics of LADs during interphase, we induced expression of the Dam–LMNB1 fusion protein for a period of 15 h to record genome–lamina interactions on the DNA. Next, the cells were collected and stained with an antibody against LMNB1 to detect current-state genome–lamina interactions with ChIC. Provided that genome–lamina interactions are dynamic, we expect that past interactions will be detected exclusively by DamID (Fig. 2a; past interactions), whereas very recently established interactions are exclusively measured by ChIC (Fig. 2a; de novo interactions).

**Extended Data Fig. 2:**
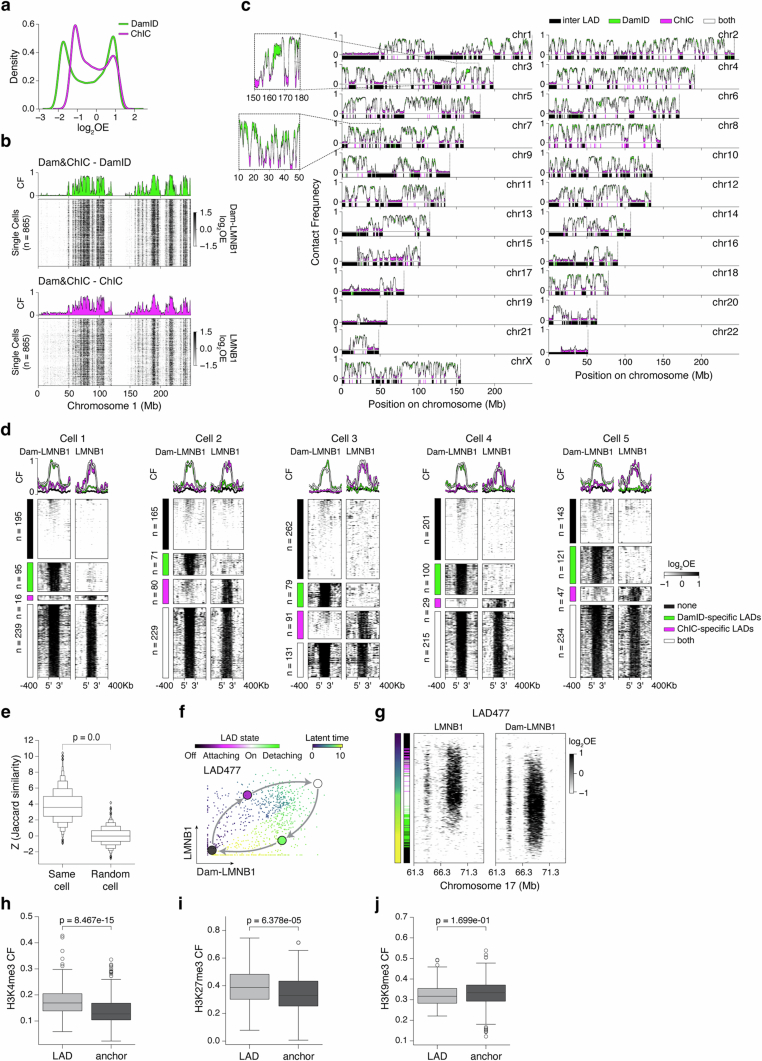
Genome–lamina interactions are differentially detected by DamID and ChIC. (**a**) Distribution of log-transformed observed over expected (OE) values of 100-Kb binned pseudobulk Dam&ChIC data. (**b**) Single-cell heatmaps showing log-transformed observed over expected (OE) LAD signal (see Methods) in 100-Kb genomic bins on chromosome 1 of either the DamID (top) or the ChIC (bottom) readout. Line plots represent Contact Frequency (CF) values. (**c**) Comparison of LAD Contact Frequency on depicted chromosomes between the DamID (green) and ChIC (magenta) measurement of Dam&ChIC. Signal detected by both DamID and ChIC is shown in white. LAD calls (see Methods) are shown below each track. (**d**) Five example single cells aligned and grouped for all bulk-based LADs according to their detection in the given cell by neither measurement (black), DamID (green), ChIC (magenta) or both (white). DamID (left) and ChIC (right) measurements are split, and respective CF values for each category are shown as line plots at the top, with a shared y axis running from 0 to 1. LAD signal is shown as log-transformed OE in 100-Kb bins. (**e**) Boxenplot of multiple quantiles of z-score normalized Jaccard similarity (see Methods) between the DamID and ChIC readouts from the same cell and any two random cells. Two-sided t-test with resulting p-values is designated. p = 0.0. (**f**) Phase portrait of LAD477 coloured by latent time inferred by scVelo^37^, describing dynamics of the LAD based on the ratio of Dam&ChIC LMNB1 data (DamID: x and ChIC: y axes). LAD states are depicted with larger dots according to the likelihood of being off (black), attaching to the lamina (magenta), on (white) or detaching from the lamina (green). (**g**) Single-cell heatmaps for LAD477, with the DamID (right) and ChIC (left) measurements split and single cells sorted according to LAD477-based latent time (leftmost bar). The middle bar at the left indicates the LAD state (see Methods). LAD signal is shown as log-transformed OE in 100-Kb bins. (**h-j**) Boxplot showing (h) H3K4me3 (p = 8.467e^−15^) (i) H3K27me3 (p = 6.378e^−05^) and (j) H3K9me3 (p = 1.699e^−01^) Contact Frequency (CF) values, quantified in 100-Kb genomic bins on anchor regions (that is LAD region where LAD starts attaching and is released; Methods) compared to the rest of the LAD. Two-sided t-test with resulting p-values are designated. Source numerical data is available in source data. Source data

We obtained high-quality single-cell LAD profiles for both Dam&ChIC measurements (Extended Data Fig. 2a,b). First, we examined the overlap in LAD detection between modalities. For this purpose, we calculated the contact frequency (CF), a metric of the fraction of cells for which a region is in contact with the lamina (Extended Data Fig. 2b)^7^. Upon visual inspection of the data, we observed that although the majority of LADs are detected by both readouts across cells, some are identified exclusively by DamID and to a lesser extent by ChIC alone (Extended Data Fig. 2c and Fig. 2b). This was confirmed by differences in the fraction of 100-kb bins that are identified by DamID, ChIC or both (Fig. 2c,d and Extended Data Fig. 2d,e). Notably, regions with intermediate CFs display the largest discrepancy between measurements, indicative of dynamic associations with the lamina (Fig. 2e). In contrast, regions with higher CFs are comparably detected between Dam&ChIC readouts (Fig. 2e), suggesting a more stable contact of these regions over time in single cells.

Given the variability of LAD detection in the present Dam&ChIC dataset, we reasoned that the ratio between DamID and ChIC in a given cell should contain information on the direction of LAD dynamics. High ChIC and low DamID signal is indicative of de novo genome–lamina interactions; conversely, low ChIC and high DamID signal implies that a LAD recently detached from the lamina. To derive these features from Dam&ChIC data, we extrapolated principles of RNA and chromatin velocity^36–38^ and used the ratio between DamID and ChIC as an approach to pseudo-order LADs across cells (Extended Data Fig. 2f). The resulting latent time^37^ orders each LAD according to its state of establishment and release over time (Fig. 2f, Extended Data Fig. 2f,g). In agreement with previous findings that genome–lamina interactions are co-ordinated in *cis*^7^, we find that neighbouring LADs change co-ordinately, with a decay over distance (Fig. 2f,g). Of note, LAD pseudo-ordering in latent time reveals polarity in the establishment of some LADs, with defined anchors of attachment (blue boxes, Fig. 2f), and a mirroring pattern of detachment (yellow boxes, Fig. 2f). In a systematic analysis, we identified the genomic position of establishment and release for all LADs and observed that LADs can establish and release either from the centre or the border of the domain (Fig. 2h). Notably, we found that LAD anchor regions have enriched A/T content (Fig. 2i), harbour fewer genes (Fig. 2j) and are depleted from hPTMs H3K4me3 (Extended Data Fig. 2h) and H3K27me3 (Extended Data Fig. 2i). H3K9me3 levels are equally enriched on anchors and the rest of the LAD (Extended Data Fig. 2j). Thus, anchors have distinct (epi)genomic features compared with non-anchor LAD regions.

Collectively, we demonstrate that Dam&ChIC enables disentangling of past and present chromatin states in the same cell and we showcase its implementation to characterize dynamics and domain organization of LADs.

### Spatial genome positioning is partially inherited upon mitosis

We next set out to implement Dam&ChIC to study the inheritance of spatial genome organization over a cell division. Previous work described repositioning of LADs in daughter cells after mitosis^35^. These observations were made through imaging LAD inheritance, but left sequence identity and the underlying patterns of genome–lamina reorganization elusive. To address this, we sought to leverage Dam&ChIC to measure retrospectively genome–lamina interactions in the same cell before and after mitosis.

**Fig. 3:**
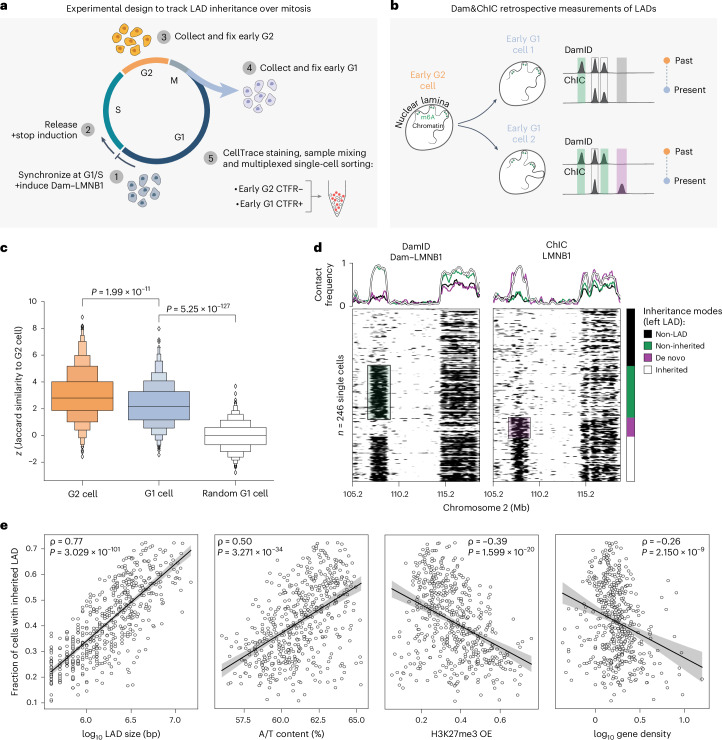
LAD inheritance over mitosis is linked to size and A/T content. **a**, Schematic of the experimental setup to track LAD mitotic inheritance (Methods), including sample multiplexing strategy. **b**, Schematic of the expected retrospective measurements when using Dam&ChIC to follow LADs over mitosis. Inherited LADs from G2 to G1 cells are expected to be captured in G1 phase by both DamID and ChIC. Non-inherited LADs are expected to be recovered only by DamID, while de novo LADs are expected to be recovered only by ChIC. **c**, Boxen plot of pairwise *z*-score normalized Jaccard similarity between DamID of G2 cell and ChIC of the same G2 cell (left), descending G1 cell (middle) or random G1 cell (right). Boxen plot shows a large number of quantiles to provide more information about the shape of the distribution. Two-sided *t*-test *P* values are *P* = 1.99 × 10^−11^ (left versus middle) and *P* = 5.25 × 10^−127^ (middle versus right). **d**, Single-cell heatmaps of DamID and ChIC measurements of Dam&ChIC, showing two LADs on Chromosome 2, one weakly inherited (left LAD) and another faithfully inherited (right LAD). Cells are grouped according to the recovery of the left LAD by DamID and/or ChIC as: non-inherited, de novo, inherited or non-LAD. The CF for each readout is plotted above per cell group. **e**, Scatter-plots for the recovery of frequency of inherited LADs (*n* = 516) against different (epi)genomic features. Y axis is the same across all plots. The black lines represent a linear regression fit with its 95% confidence interval in the shaded area. From left to right Pearson Correlation coefficients (ρ) are 0.77, 0.50, −0.39 and −0.26 and *P* values of two-sided testing for non-correlation are 3.029 × 10^−101^, 3.271 × 10^−34^, 1.599 × 10^−20^ and 2.150 × 10^−09^. Source numerical data are available in source data. Source data

**Extended Data Fig. 3:**
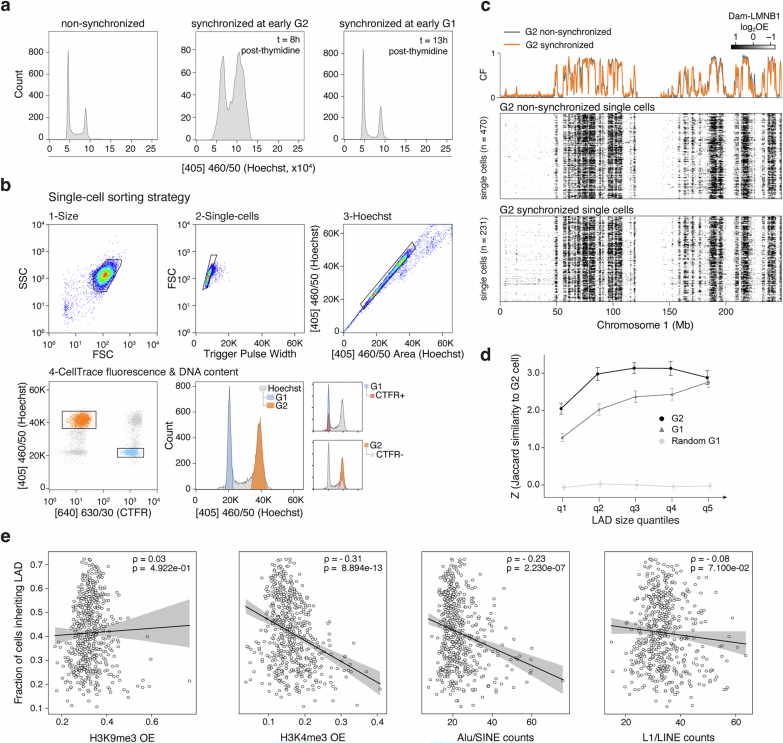
Technical validations of the LAD inheritance data. **(a)** Hoechst-based DNA content distributions of the non-synchronized control and the synchronized in G2 and synchronized in G1 samples prior to sample mixing and single-cell sorting for Dam&ChIC (**b**) Single-cell sorting strategy (see Methods), including the used gates based on size, singlets, Hoechst staining and CellTrace stainings that were used for multiplexing. CellTrace Far Red is noted as CTFR. (**c**) Single-cell heatmaps and corresponding Contact Frequency (CF) quantifications, comparing G2 synchronized with G2 non-synchronized cells. (**d**) Z-score normalized Jaccard similarity between DamID and ChIC readouts within G2 cells, within G1 cells, and between random G1 cells, across different LAD size quantiles. (**e**) Scatter-plots for the recovery of frequency of inherited LADs (n = 516) against different (epi)genomic features. Y axis is the same across all plots. The black lines represent a linear regression fit with its 95% confidence interval in the shaded area. From left to right, the Pearson Correlation coefficient (ρ) are 0.03, −0.31, −0.23 and −0.08. P-values of two-sided testing for non-correlation are 4.922e^−01^, 8.894e^−13^, 2.230e^−07^ and 7.100e^−02^. Source numerical data is available in source data. Source data

We synchronized KBM7 cells in G1/S using a double thymidine block and expressed Dam–LMNB1 concurrently with the last thymidine incubation (Fig. 3a and Methods). We collected cells in G2 and early G1 phase (Extended Data Fig. 3a) and utilized a fluorescence-based sample multiplexing strategy for single-cell sorting^39,40^ (Extended Data Fig. 3b and Methods). The experimental setup ensures that Dam–LMNB1 is still present during S and G2 to label and record G2 LADs (Extended Data Fig. 3c) and cell collection 1 h in G1 prevents de novo accumulation of ^m6^A as shown previously^35^. Therefore, in early G1 cells, LADs inherited through mitosis will be detected by both DamID and ChIC (Fig. 3b, cells 1 and 2, uncoloured boxes), non-inherited LADs (those that reposition away from the lamina after mitosis), will be exclusively recovered by DamID (Fig. 3b, cells 1 and 2, green boxes) and de novo established LADs exclusively by ChIC (Fig. 3b, cell 2, purple box).

To validate our synchronization and induction approach, we first confirmed that G2-synchronized cells display typical LAD profiles (Extended Data Fig. 3c). We then computed the *z*-score normalized Jaccard index, to measure the pairwise similarity between DamID and ChIC measurements (Methods) between: (1) DamID and ChIC of the same cell in G2; (2) DamID and ChIC of the same cell in early G1; or (3) DamID and ChIC of random cells in G1. We observed a significant drop in similarity between DamID and ChIC LAD profiles in G1 compared with G2 cells, corroborating previous microscopy findings of LAD reshuffling after mitosis^35^. Yet, the similarity in G1 cells is still considerably higher than what is observed when comparing random cells (Fig. 3c). This suggests that LADs are partially inherited from G2 to early G1.

Notably, the degree of inheritance varies extensively between LADs (Fig. 3d), with some domains showing considerable heterogeneity (Fig. 3d, left LAD), while others display stable inheritance across cells (Fig. 3d, right LAD). We therefore wondered what features endow LADs with higher propensity to be inherited through mitosis than others. We found size and A/T content of LADs positively correlating with inheritance (Fig. 3e, left-most panels), which is further verified by increased similarity in the G1 population for large LAD sizes (Extended Data Fig. 3d). H3K27me3 enrichment and gene density show a negative correlation with inheritance (Fig. 3e, right-most panels), whereas H3K9me3, H3K4me3, SINE and LINE content show weak negative or no correlation with LAD inheritance (Extended Data Fig. 3e). These data indicate that larger, A/T-rich, gene- and H3K27me3-poor LADs are more faithfully inherited upon mitosis, corroborating previous findings that LADs in single cells are organized through multivalent contacts of large stretches of A/T-rich DNA^7^.

In summary, we show the ability of Dam&ChIC to capture chromatin transitions over a cell division, providing a unique framework to obtain insights into epigenetic inheritance.

### The inactive X chromosome detaches from the nuclear lamina

We next sought to utilize Dam&ChIC to dissect the interplay between chromatin features during XCI, an ideal model to study heterochromatin formation. During XCI, dramatic remodelling results in transcriptional repression of the inactive X chromosome (Xi), to ensure dosage compensation of X-linked genes between males (XY) and females (XX)^41–43^. Heterochromatin formation initiates by coating of the Xi by the long noncoding RNA Xist^44–46^ and subsequent deposition and spreading of Polycomb hPTMs H2AK119Ub and H3K27me3^47–50^. While remodelling of the hPTM landscape on the Xi has been extensively studied, its nuclear positioning remains unclear. Previous studies suggest that the Xi associates with different nuclear compartments, such as the nucleolus or the lamina^51–57^. Whether this interaction is dynamic and how it is linked to other hPTMs has remained ambiguous.

**Fig. 4:**
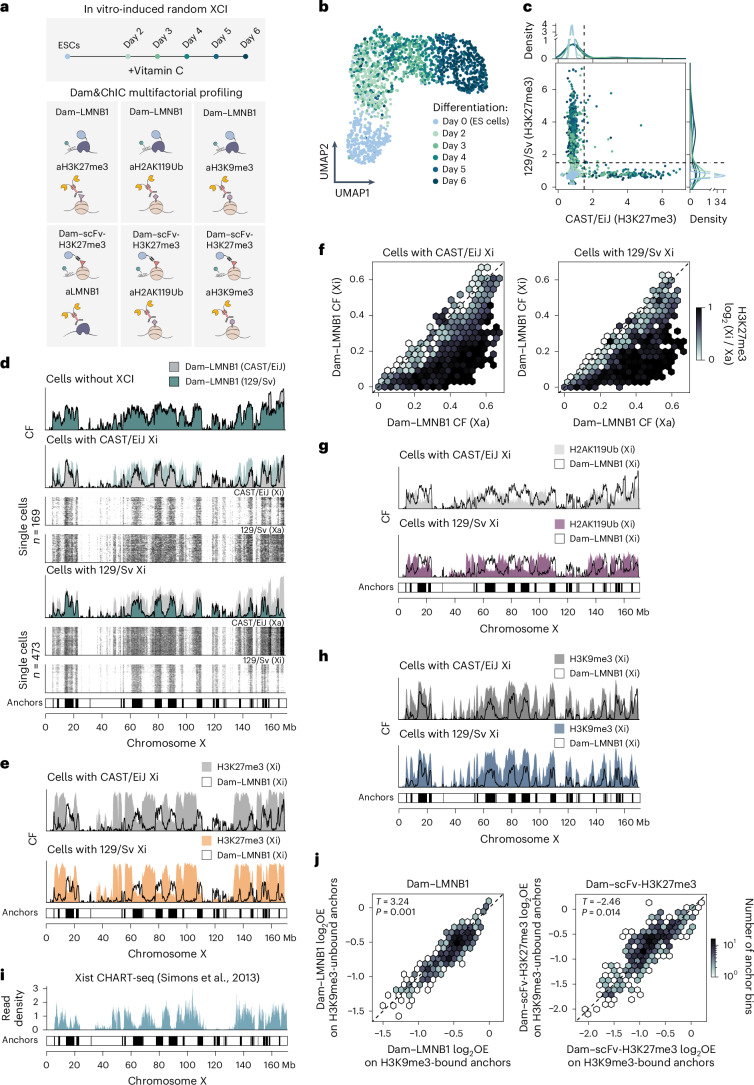
Genome–lamina interactions are lost at Polycomb domains on the inactive X chromosome. **a**, Schematic of various Dam&ChIC combinations obtained during vitamin C differentiation. **b**, UMAP (*n* = 1,656 cells) based on H3K27me3 levels on autosomal genes of Dam–LMNB1/H3K27me3-profiled cells, coloured by differentiation day. **c**, Scatter-plot of allelic X-chromosome H3K27me3 OE levels per single cell in the Dam–LMNB1/H3K27me3 dataset, coloured by differentiation day. Dashed lines indicate manually set threshold used to classify cells as ‘no XCI’, ‘CAST/EiJ inactive’, ‘129S1/Sv inactive’ or ‘Undetermined’. **d**, CF values of Dam–LMNB1 on either allele for cell categories defined in **c**, along the entire X chromosome. *y* axes in CF plots run from 0 to 1. For categories that undergo XCI, single-cell heatmaps of the active (Xa) and inactive (Xi) X chromosome are shown below, with respective cell numbers. Plotted cells span all differentiation time points that XCI takes place, based on **c**. Bottom bar shows calls of the anchors, defined as LMNB1-retaining H3K27me3-depleted regions on Xi (S4m, bottom right quadrant). **e**, CF values of Dam–LMNB1 and corresponding H3K27me3 on either allele in the inactive state for cell categories defined in **c** along the entire X chromosome. *y* axes in CF plots run from 0 to 1. **f**, Hexbin plot of Dam–LMNB1 CF values in cells where the indicated allele is inactivated (left: CAST/EiJ, right: 129S1/Sv). For each LAD, average CF is shown for the Xa versus the Xi allele. Hexbins are coloured based on log-transformed enrichment of H3K27me3 (Xi/Xa). **g**, CF values of Dam–LMNB1 and H2AK119Ub on either allele in the inactivated state, as in **e**. **h**, CF values of Dam–LMNB1 and H3K9me3 on either allele in the inactivated state, as in **e**. **i**, Published Xist CHART-seq data derived from differentiated mouse ES cells (d7)^66^ for the X chromosome. The *y* axis is expressed as scaled read density values. **j**, Comparison of Dam–LMNB1 (left) and Dam–scFv-H3K27me3 (right) levels between cells in which NL anchors are H3K9me3-bound (*x* axis) or unbound (*y* axis). Hexbins are coloured by anchor abundance. Two-sided *t*-test with resulting *P* values are *P* = 0.001 (left) and *P* = 0.014 (right). Source numerical data are available in source data. Source data

**Extended Data Fig. 4:**
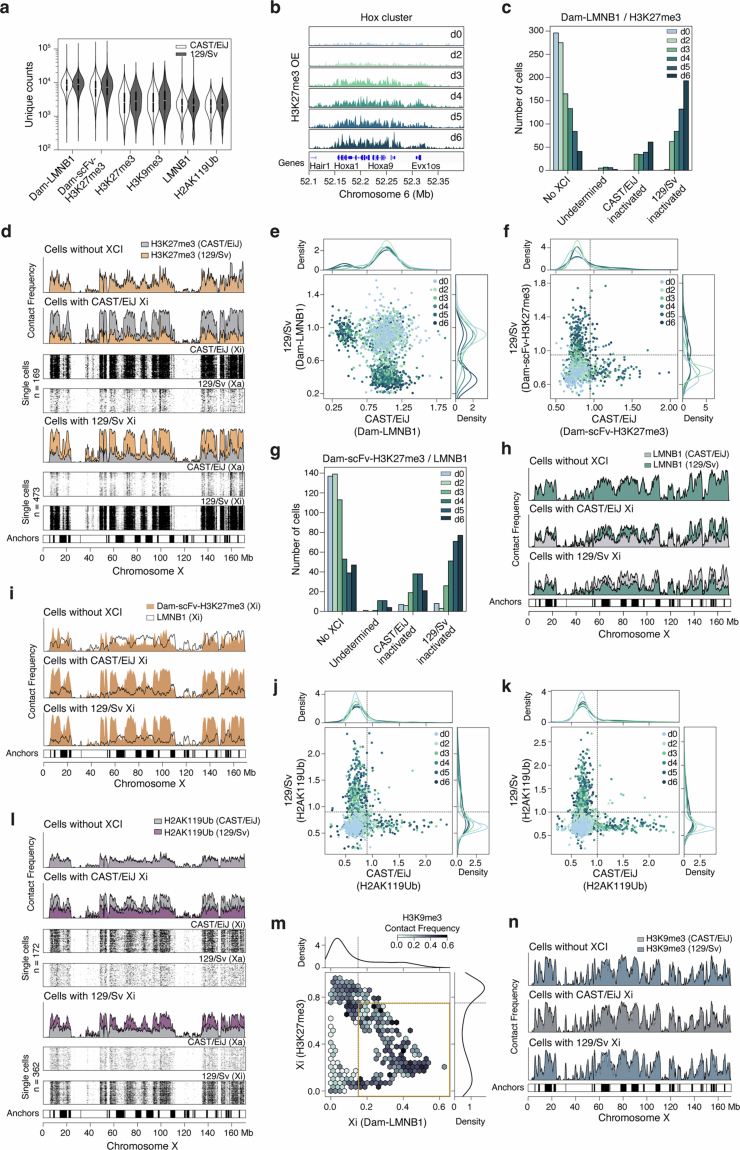
Detaching regions rich in polycomb marks are interspersed by H3K9me3-rich anchors. (**a**) Violin plots with number of unique counts per cell and allele for all readouts obtained with Dam&ChIC during differentiation. (**b**) Observed over expected (OE) levels of H3K27me3 over the Hoxa gene cluster on Chromosome 6, divided by experimental time point. (**c**) Number of cells in each differentiation day for all categories defined in Fig. 4c. Undetermined cells have high levels of H3K27me3 on both alleles. (**d**) Contact Frequency (CF) values of H3K27me3 on either allele for cell categories defined in Fig. 4c, along the entire X chromosome. Y axes in CF plots run from 0 to 1. Single-cell heatmaps of the active (Xa) and inactive (Xi) X chromosome are shown for categories that undergo XCI, with respective cell numbers. Bottom bar shows calls of the anchors. (**e**) Scatter-plot of allelic Dam-LMNB1 levels per single cell in the Dam-LMNB1/H3K27me3 dataset. (**f**) Scatter-plot of allelic Dam-scFv-H3K27me3 levels per single cell in the Dam-scFv-H3K27me3/LMNB1 dataset. Dashed lines indicate the manually set threshold used to classify cells as no XCI, CAST/EiJ inactive, or 129S1/Sv inactive. (**g**) Similar to (**c**), based on Dam-scFc-H3K27me3/LMNB1 data. (**h**) CF values of LMNB1 on either allele for the cell categories defined in (**f**). Y axes run from 0 to 1. (**i**) CF values of LMNB1 and corresponding Dam-scFv-H3K27me3 values on either allele in the inactive state for cell categories defined in (**f**). Y axes run from 0 to 1. (**j**) Scatter-plot of allelic H2AK119Ub levels per single cell in the Dam-LMNB1/H2AK119Ub dataset. Dashed lines indicate manually set threshold used to classify cells like in (**f**). (**k**) Similar to (**j**) for the Dam-scFv-H3K27me3/H2AK119Ub dataset. (**l**) Similar to (**d**) for H2AK119Ub. Data derives from the Dam-LMNB1/H2AK119Ub dataset. (**m**) Hexbin plots of Dam-LMNB1 (x axis) and H3K27me3 (y axis) CF for all X chromosome bins. Each hexbin is a collection of X chromosome bins. Dashed lines indicate manually set thresholds used to classify regions as anchors (yellow box; bottom right quadrant). Colour projection on hexbins is average H3K9me3 CF. (**n**) Similar to (**h**), for H3K9me3. Data derives from the Dam-LMNB1/H3K9me3 dataset. Y axes run from 0 to 1. Source numerical data is available in source data. Source data

To address this, we induced random XCI in vitro^58,59^, by differentiating two female hybrid (CAST/Eij × 129/Sv) mouse embryonic stem (ES) cell lines expressing Dam–LMNB1^60^ or Dam–scFv-H3K27me3 (ref. ^27^) with vitamin C (Fig. 4a). We obtained Dam&ChIC datasets of Dam–LMNB1/H3K27me3 (*n* = 1,656 cells), Dam–LMNB1/H3K9me3 (*n* = 573 cells), Dam–LMNB1/H2AK119Ub (*n* = 1,213 cells), Dam–scFv-H3K27me3/LMNB1 (*n* = 921 cells), Dam–scFv-H3K27me3/H3K9me3 (*n* = 497 cells) and Dam–scFv-H3K27me3/H2AK119Ub (*n* = 1,404 cells) at sequential time points between day 0 and 6 of differentiation (Fig. 4a). Dam&ChIC data were split on alleles based on single-nucleotide polymorphisms (Methods), yielding consistently high numbers of unique counts per allele for all measurements (Extended Data Fig. 4a).

First, we examined the Dam–LMNB1/H3K27me3 dataset. The H3K27me3 readout was used to estimate both the progression of differentiation, and the progression of XCI. Dimensionality reduction using autosomal H3K27me3 enrichment revealed the expected gradual transitioning of cells over differentiation time (Fig. 4b), further corroborated by gradual accumulation of H3K27me3 on the Hox cluster locus (Extended Data Fig. 4b). We observed strong allelic H3K27me3 enrichment, which we used to categorize cells according to which X allele was inactivated (Fig. 4c and Extended Data Fig. 4c,d).

Notably, we found extensive depletion of LMNB1 signal across single cells, which is specific to the Xi, but does not occur on the Xa (Fig. 4d and Extended Data Fig. 4e). This is confirmed in the reciprocal control dataset measuring H3K27me3 with DamID and LMNB1 with ChIC (Extended Data Fig. 4f–h). Loss of genome–lamina interactions extends through megabase-sized regions across the entire X chromosome. These are interspersed by regions retaining lamina interactions (henceforth referred to as ‘anchors’), covering roughly 29% of the Xi (Fig. 4d, bottom bar and Extended Data Fig. 4m). Of note, detaching regions are highly enriched with H3K27me3, as opposed to anchors that are devoid of H3K27me3 (Fig. 4e,f and Extended Data Fig. 4i,m). We further wondered whether detaching regions coincide with enrichment of H2AK119Ub^49^, which shows overlapping distributions and is deposited by Polycomb complexes before H3K27me3 (refs. ^50,61–63^). Using our Dam–LMNB1/H2AK119Ub and Dam–scFv-H3K27me3/H2AK119Ub datasets, we found a strong allelic enrichment of H2AK119Ub on the X chromosome over differentiation (Extended Data Fig. 4j–l), and confirmed that, similar to H3K27me3, detaching regions are enriched with H2AK119Ub (Fig. 4g). Altogether, this data indicates that spatial nuclear repositioning of the Xi is linked to accumulation of Polycomb hPTMs H3K27me3 and H2AK119Ub.

Genomic regions with constitutive genome–lamina interactions are generally enriched in repressive hPTMs H3K9me2/3 (ref. ^26^). We therefore wondered if anchors are H3K9me3-enriched. Indeed, our Dam–LMNB1/H3K9me3 and Dam–scFv-H3K27me3/H3K9me3 datasets show a high enrichment of H3K9me3 on anchors (Fig. 4h and Extended Data Fig. 4m), consistent with previous observations of non-overlapping patterns of enrichment between H3K9me3 and H3K27me3 along the Xi^64,65^. Notably, anchors are devoid of Xist spreading, as measured previously by CHART-seq^66^ (Fig. 4i), and they pre-exist in cells that did not yet undergo XCI (Extended Data Fig. 4n). Finally, we wondered whether anchors are differentially used across cells, and if this is linked to the pre-existing H3K9me3 state. Therefore, we quantified LMNB1 and H3K27me3 levels for H3K9me3-enriched or depleted anchors across single cells, and found that anchors enriched with H3K9me3 are more likely to interact with the lamina and less likely to be H3K27me3-rich (Fig. 4j). This suggests that X chromosome regions lacking H3K9me3 are predetermined to detach from the lamina and accumulate H3K27me3 during XCI.

In summary, Dam&ChIC provides allelically-resolved single-cell maps of genome–lamina interactions and heterochromatic hPTMs from the same cell. We show that large X-chromosome regions detach from the lamina, and instead become strongly enriched with Polycomb hPTMs during XCI. Regions resistant to detachment are refractory to Xist spreading and Polycomb deposition and coincide with pre-existing H3K9me3 domains.

### Detachment precedes Polycomb domain formation on the Xi

**Fig. 5:**
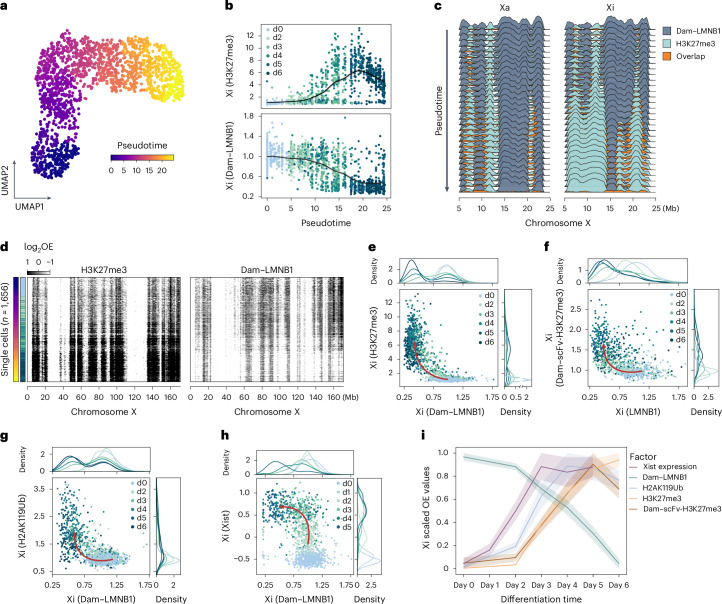
Detachment initiates before spreading of Polycomb, but follows Xist expression. **a**, Same UMAP as in Fig. 4b, based on H3K27me3 levels on autosomal genes, coloured by Monocle3-inferred pseudotime. **b**, Average OE H3K27me3 (top) and Dam–LMNB1 (bottom) levels (*y* axis) across pseudotime (*x* axis) for the inactive X. Cells are coloured according to differentiation day. **c**, CF of Dam–LMNB1, H3K27me3 and their overlap for groups of 100 cells progressively slid along pseudotime (*y* axis) for a dynamic region of the inactive X (right) and the active (left). **d**, Single-cell heatmaps (*n* = 1,656) for H3K27me3 (left) and Dam–LMNB1 (right) for the entire inactive X. Cells (*y* axis) are ordered along pseudotime. The colour scale indicates log-transformed OE values for each readout. **e**, Scatter-plot of the average inactive X (Xi) Dam–LMNB1 (*x* axis) and H3K27me3 (*y* axis) OE levels. Cells are coloured according to differentiation day. Distributions above and on the right show average density per day. Arrow is manually drawn based on directionality of cells over differentiation days. **f**, Same as **e**, but with the inverse readout: Dam–scFc-H3K27me3 (*y* axis) / LMNB1 (*x* axis). **g**, Same as **e**, for Dam–LMNB1 (*x* axis) / H2AK119Ub (*y* axis). **h**, Same as **e**, for Dam–LMNB1 (*x* axis)/Xist expression (*y* axis). Xist levels are represented as transcripts per million. **i**, Summary of the order of events profiled during vitamin C differentiation, with each plotted as average OE values per differentiation day. 95% confidence interval is shown in the shaded area. Source numerical data are available in source data. Source data

As genome–lamina interactions and Polycomb hPTMs show a mutually exclusive pattern on the Xi, we were prompted to dissect the temporal relationship between these events. To identify the onset of detachment and H3K27me3 accumulation, we ordered cells along the differentiation trajectory by inferring pseudotime^67,68^ (Fig. 5a). Detachment seems to coincide with the emergence of H3K27me3 around day 3 of differentiation (Fig. 5b), with a seemingly concurrent onset of both events based on progressive pseudobulk profiles (Fig. 5c) and single cells (Fig. 5d). In addition, while H3K27me3 domains are progressively expanding outwards from sites already present in undifferentiated cells^69^, LADs gradually shrink along the Xi (Fig. 5c).

**Extended Data Fig. 5:**
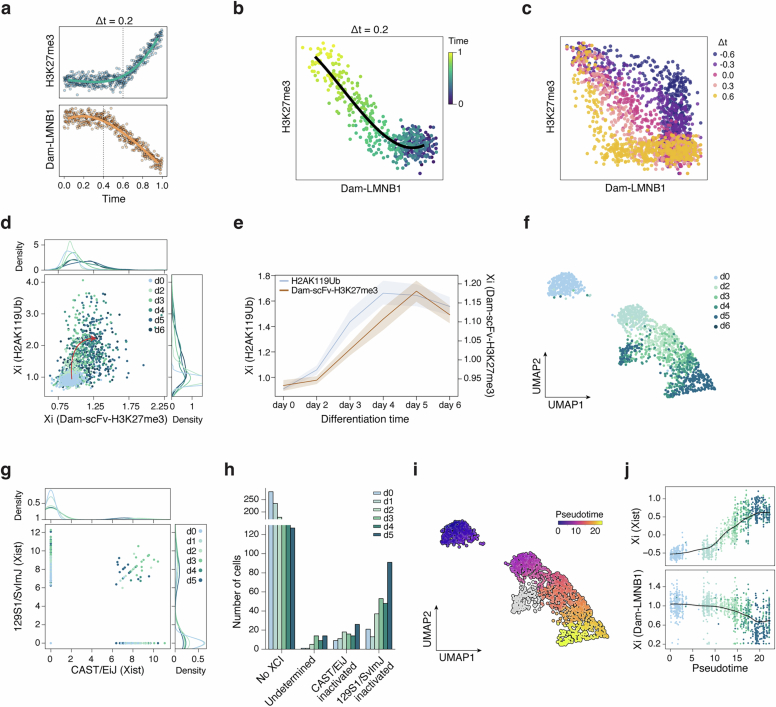
Sequential chromatin remodelling during XCI. (**a**) Simulated single-cell H3K27me3 (top) and Dam-LMNB1 (bottom) values, with controlled onset of both dynamics to enable comparison of their relative timing. Values are generated by randomly sampling from a log-normal distribution and the onset of dynamics is simulated by randomly sampling from a Poisson distribution. Simulated time difference (Δt) is 0.2. (**b**) Scatter-plot of simulated Dam-LMNB1 (x axis) and H3K27me3 (y axis) values from (**a**). Cells are coloured according to simulated time. (**c**) Same as (**b**), with cells following and coloured according to progressive Δt values. (**d**) Scatter-plot of the average inactive X (Xi) Dam-scFv-H3K27me3 (x axis) and H2AK119Ub (y axis) observed over expected (OE) levels. Cells are coloured according to differentiation day. Distributions above and on the right show average density per day. Arrow is manually drawn based on directionality of cells over differentiation days. (**e**) Lineplot showing Dam-scFv-H3K27me3 & H2AK119Ub dynamics on the Xi over differentiation. Values plotted as average OE with the 95% confidence interval in the shaded area. (**f**) UMAP (n = 1427 cells) based on transcriptomic data of scDam&T-seq, coloured by differentiation day. (**g**) Scatter-plot of allelic Xist expression per single cell, coloured by differentiation day. (**h**) Number of cells in each differentiation day for all categories of cells based on allelic Xist expression in (**g**). (**i**) Same UMAP as in (**f**) coloured by Monocle3-inferred pseudotime. (**j**) Xist expression (top) and Dam-LMNB1 (bottom) levels (y axis) across pseudotime (x axis) over the Xi. Xist levels are represented as transcripts per million and Dam-LMNB1 levels as OE. Cells are coloured according to differentiation day. Source numerical data is available in source data. Source data

To resolve the precise chronology between detachment and H3K27me3 deposition, we scattered for each cell along XCI their Dam–LMNB1 and H3K27me3 levels on the Xi. This confirmed their inverse relationship, and revealed that reduction of Dam–LMNB1 levels occurs before H3K27me3 accumulation, in cells belonging to early differentiation time points (Fig. 5e, arrow). To substantiate our approach, we used simulations with controlled temporal ordering of the two readouts and confirmed that the observed left-upward mobility in Fig. 5e corresponds to the scenario where detachment precedes H3K27me3 accumulation (Extended Data Fig. 5a–c). Reassuringly, we identify the same pattern in the inverse Dam&ChIC experiment (Dam–scFv-H3K27me3/LMNB1), detecting an even earlier onset of detachment (Fig. 5f). Similar analyses showed that detachment also precedes H2AK119Ub accumulation on the Xi (Fig. 5g), and that H2AK119Ub precedes H3K27me3, confirming previous reports (Extended Data Fig. 5d,e)^50,61–63^. Despite the fact that both Polycomb hPTMs start accumulating on the Xi after the onset of detachment, these processes continue to consolidate over time.

As detachment seems to happen earlier than formation of Polycomb domains, we next set out to characterize its temporal relationship with expression of Xist, which is known to interact with and recruit Polycomb complexes to the X chromosome^61,70,71^. To this end, we performed scDam&T-seq^29,33^ during vitamin C differentiation and measured genome–lamina contacts and transcriptomes from the same cells (Extended Data Fig. 5f–j). Unlike H2AK119Ub and H3K27me3 (Fig. 5e–g), detachment of the Xi from the lamina follows Xist expression (Fig. 5h,i).

Taken together, we demonstrate that the loss of genome–lamina interactions is an early event in mouse random XCI, temporally linked to Xist and Polycomb. These data highlight the ability of Dam&ChIC to uncover the hierarchical order of events during chromatin remodelling with unprecedented detail.

### Detachment of the Xi occurs upon mitotic exit

**Fig. 6:**
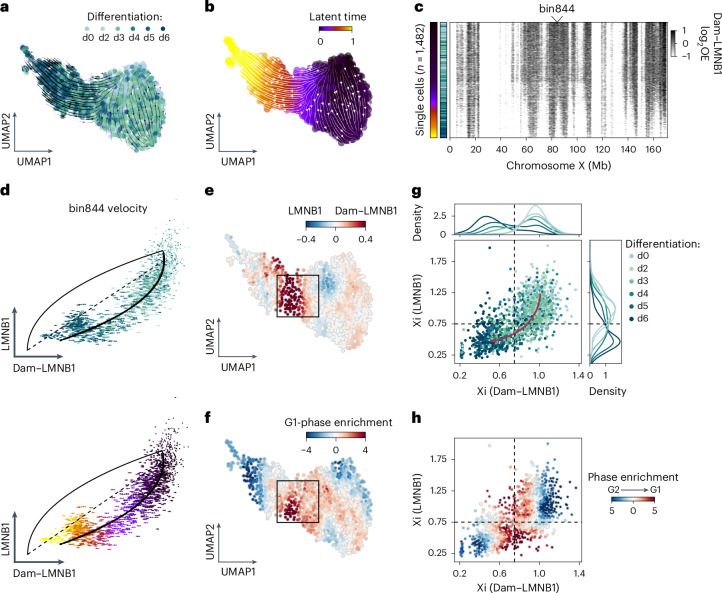
Retrospective measurements link detachment of the Xi to cell cycle phase. **a**, UMAP (*n* = 1,482 cells) based on Dam–LMNB1 levels in 100-kb-bins of the Xi coloured according to differentiation day. Streamlines on top of UMAP represent chromatin velocities inferred by the ratio of Dam–LMNB1 and LMNB1 in 100-kb-bins of the Xi, using scVelo^29^. **b**, Same UMAP as in **a**, but with cells coloured according to scVelo-inferred latent time, which is purely based on Dam–LMNB1/LMNB1 dynamics, not on the underlying UMAP. **c**, Single-cell heatmap showing log-transformed Dam–LMNB1 OE values on the entire inactivating X chromosome, with cells ordered along the inferred latent time shown in **b**. **d**, Scatter-plots showing the ratio of Dam–LMNB1 (*x* axis) and LMNB1 (*y* axis) in each cell for X chromosome bin 844 (marked in **c**). Length of arrows reflect the Dam–LMNB1/LMNB1 velocity of bin844 in that cell. Arrows are coloured according to differentiation day (top) or latent time (bottom). (**e**) Same UMAP as in **a**, coloured according to the ratio between Dam–LMNB1 and LMNB1. **f**, Same UMAP as in **a**, coloured according to the *z*-score normalized G1 cell cycle-phase enrichment, which is calculated with a *k*-nearest neighbours approach over the neighbourhood a cell belongs to (Methods). **g**, Scatter-plot of average Dam–LMNB1 (*x* axis) and LMNB1 (*y* axis) OE levels on the Xi. Each dot represents the Xi in one cell, coloured according to differentiation day. Distributions above and on the right show average density per day. Arrow is manually drawn based on directionality of cells over differentiation days. **h**, Same scatter-plot as in **g**, with points coloured according to the ratio between *z*-score-normalized G1 and G2 cell-cycle phase enrichment (Methods). Cell-cycle data are available in Supplementary Table 3. Source data

**Extended Data Fig. 6:**
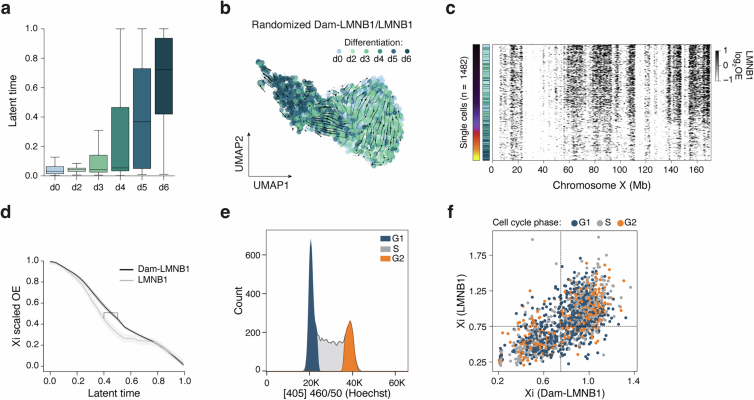
Velocity-based ordering pinpoints detachment during XCI. (**a**) Distribution of cells from Vitamin C differentiation days across latent time defined in Fig. 6b. (**b**) Same UMAP as in (**a**), based on Dam-LMNB1 levels in 100-Kb-bins of the Xi, with streamlines representing chromatin velocities of randomly coupled Dam-LMNB1 and LMNB1 readouts per cell. (**c**) Single-cell heatmap, showing log-transformed LMNB1 observed over expected (OE) values on the entire inactivating X-chromosome, with cells ordered along the inferred latent time shown in (**b**). (**d**) Average Dam-LMNB1 (black) and LMNB1 (grey) OE levels of the inactive X chromosome along latent time. Gap marks the moment of highest genome–lamina dynamics on the Xi. (**e**) Example Hoechst profile of 380 sorted single cells from the Dam-LMNB1/LMNB1 Vitamin C experiment, showing their DNA content and respective cell-cycle stage. (**f**) Same scatter-plot as in Fig. 6g with raw cell cycle-phase annotations determined based on Hoechst values. Cell-cycle data is available in Supplementary Table 3. Source numerical data is available in source data. Source data

Prompted by the increased LAD reorganization we saw in KMB7 cells upon mitosis, we asked whether detachment of the Xi is cell cycle-related. To answer this question, we profiled genome–lamina interactions with both readouts of Dam&ChIC (Dam–LMNB1/LMNB1) during vitamin C differentiation. This dataset allows distinguishing past from present genome–lamina interactions in the same cells. Using solely inactive X-chromosome data, we estimated the ratio between past and present genome–lamina interactions to infer chromatin velocity and latent time over the differentiation trajectory (Fig. 6a,b and Methods). This analysis recapitulates the direction of differentiation, as shown by the progressive distribution of cells from different experimental time points across latent time (Extended Data Fig. 6a). Reassuringly, the velocity model fails to deduce directionality when randomly coupled DamID and ChIC readouts are used (Extended Data Fig. 6b). Ordering of single-cell Xi profiles based on latent time shows the typical loss of genome–lamina interactions for both DamID and ChIC (Fig. 6c,d and Extended Data Fig. 6c). When comparing the dynamics of genome–lamina interactions measured by DamID and ChIC across latent time, we recover a temporal delay between both measurements, reflecting the rapid detachment of the Xi from the lamina (Extended Data Fig. 6d).

To pinpoint whether detachment of the Xi is linked to a specific cell-cycle state, we used Hoechst-based DNA content data, which were measured for each cell during single-cell sorting (Extended Data Fig. 6e). Single cells displaying the highest ratio between past (Dam–LMNB1) and present (LMNB1) genome–lamina interactions on the Xi (Fig. 6e), show the highest G1 phase enrichment (Fig. 6f and Methods). Moreover, when projecting cell-cycle phase enrichment over the genome–lamina interaction dynamics of single cells (Fig. 6g,h and Extended Data Fig. 6f), we observe that detachment of the Xi from the lamina coincides with the transition from G2 to G1 phase. Taken together, the co-occurrence of lamina detachment and entry into G1 phase suggests that genome–lamina interactions of the Xi might be lost over mitosis.

## Discussion

### Advantages and limitations of Dam&ChIC

We developed a chromatin-profiling technology for simultaneously profiling two distinct chromatin features at high resolution, or for measuring retrospectively chromatin-state transitions over time in the same cell. Unlike other methods, Dam&ChIC does not involve in situ tagging of the genome, which essentially allows measurements of only two chromatin features at a time, yet with unprecedented sensitivity. It is a suitable method to examine the interplay between features that range from euchromatic to constitutively heterochromatic, and interrogate co-ordinated gene regulation at the scale of large domains down to individual genes and promoters.

The challenge with any DamID-based methodology is the requirement to engineer and optimize cell lines, which is a substantial time investment. However, many cell lines have already been established besides those used in this study, such as for DNA repair proteins^72^ single-chain antibodies and engineered chromatin reader domains^27^. We expect the repertoire of Dam–POI fusions to continue expanding, broadening the applicability of the method.

Of note, as a plate- and FACS-based approach, Dam&ChIC is ideal for medium-throughput experiments, providing flexibility at a low cost compared with high-throughput approaches. Additionally, using the FACS-recorded data on various cellular characteristics, it is possible to enrich rare cells of interest based on known cell (surface) markers^30^, multiplex samples by fluorescence-based hashing^39,40^, or integrate cell-cycle information in single-cell analyses. We anticipate that future iterations of Dam&ChIC that render it compatible with combinatorial indexing or microfluidics will substantially increase its throughput.

### Recording chromatin dynamics over time with Dam&ChIC

Tracking chromatin changes over time in the same cell has been an attractive yet challenging concept. Such technologies would enable the study of poorly understood aspects of epigenetic regulation; for example, how past chromatin changes affect present cellular states. With Dam&ChIC we set first steps in this direction, by experimentally integrating the cumulative past chromatin information recorded by DamID, with snapshot information measured by ChIC. Currently, Dam&ChIC is limited to recording short-term chromatin dynamics, given the absence of a maintenance machinery in eukaryotes that propagates the ^m6^A mark during DNA replication. Consequently, the time window to study chromatin dynamics with Dam&ChIC currently spans between two replication cycles. The present Dam&ChIC protocol is therefore very suitable to disentangle the role of chromatin dynamics in cell cycle-related phenomena, or other relatively rapid chromatin changes. A candidate to aid propagation of the exogenous ^m6^A mark over many cellular generations is the Dam mutant L122A, which has been shown to transfer ^m6^A only on hemi-methylated DNA^73^, similar to DNMT1 for DNA CpG methylation. In the future, improved engineered versions of such exogenous methyltransferases could make tracking of chromatin dynamics over multiple cell generations feasible.

### Sequential chromatin remodelling during XCI

We used Dam&ChIC to explore the interplay of chromatin features, with a main focus on nuclear localization of the X chromosome during XCI. Leveraging both multifactorial and retrospective measurements, we assembled a set of observations that shed light on the timing and hierarchies of factors underlying heterochromatin formation. We show that upon Xist expression, the Xi undergoes extensive loss of genome–lamina interactions, initiated after mitotic exit in G1 phase. Detachment is followed by Polycomb-deposited H2AK119Ub enrichment, which precedes, as previously shown, H3K27me3 enrichment.

Previous microscopy-based studies have described the nuclear periphery or the nucleolus as the preferred nuclear locations of the Xi^53,54^. More recent findings indicated that the Xi is actively recruited to the NL through interactions of Xist with the lamin B1 receptor (LBR)^57^. Contrary to that, we find extensive lamina detachment across megabase regions on the Xi, which occurs early during XCI, following Xist expression. However, some genome–lamina interactions are retained in H3K9me3-rich anchor regions that seem resistant to spreading of Xist and are present before XCI initiation. Given this order of events, our data support previous findings that XCI initiation is not determined by artificial tethering of the X chromosome to the NL^74^. Moreover, it is possible that detaching regions may instead form interactions with the nucleolus.

The observation that detachment occurs on domains with high Polycomb hPTM enrichment, suggests an antagonistic interplay between genome–lamina interactions and Polycomb during XCI. A similar phenomenon has recently been described in two unrelated systems, namely K562 cells and preimplantation embryos, where depletion of H3K27me3 led to increased genome–lamina interactions^60,75^. Despite the clear precedence of detachment over Polycomb spreading, we find that the two processes are not sequentially completed. Instead, both gradually consolidate over XCI, indicative of potential interplay whereby upon initial detachment, deposited Polycomb marks may further hinder genome–lamina interactions. Polycomb complexes are recruited to the X chromosome by Xist, with a strong interdependency in spreading^76^, whereas Xist expression precedes detachment. A compelling avenue of future research could be to elucidate whether detachment is functionally linked to spreading of Xist, Polycomb and transcriptional silencing.

## Conclusion

Several aspects of chromatin regulation have been poorly understood due to the limitations posed by conventional technologies. Dam&ChIC introduces unique possibilities to investigate in depth the interplay between diverse chromatin factors, and to track retrospectively temporal changes within the same cell. Our single-cell multifactorial chromatin data and retrospective analyses elucidate aspects of chromatin regulation that are involved in the inheritance of chromatin upon mitosis and the formation of heterochromatin during XCI. These properties establish Dam&ChIC as a tool in the rapidly growing set of single-cell chromatin technologies.

## Methods

Methods developed or utilized in the present work are also available on protocols.io^77^.

### Dam–POI cell lines

KBM7 cells expressing Dam–LMNB1 or untethered Dam under the control of the destabilizing domain system were generated previously using lentiviral transductions^7^. Female mouse F1 hybrid Cast/EiJ × 129/Sv ES cells were a kind gift from the J. Gribnau laboratory. The Dam–LMNB1 or Dam–scFv-H3K27me3 constructs were previously integrated in these cells using CRISPR targeting^27,60^. Both ES cell lines express the Dam–POI under the control of the AID degron system. In Dam–scFv-H3K27me3 ES cells, expression of the Dam fusion protein is additionally controlled by tamoxifen-inducible nuclear translocation via the oestrogen receptor.

### Cell culture

All cell lines were cultured in a humidified chamber at 37 °C in 5% CO_2_ and regularly tested for *Mycoplasma*. Human haploid KBM7 cells were cultured in suspension in IMDM (Gibco, 12440053) supplemented with 10% FBS (Sigma, F7514) and 1% pen–strep (Gibco, 15140122). Mouse F1 hybrid ES cells were cultured on a monolayer of irradiated primary mouse embryonic fibroblasts in CM+/+, as described previously^27^. CM+/+ consists of G-MEM (Gibco, 11710035) supplemented with 10% FBS, 1% pen–strep, 1× GlutaMAX (Gibco, 35050061), 1× non-essential amino acids (Gibco, 11140050), 1× sodium pyruvate (Gibco, 11360070), 0.1 mM β-mercaptoethanol (Sigma, M3148) and 1,000 U ml^−1^ ESGROmLIF (EMD Millipore, ESG1107). Before differentiation, mouse ES cells were passaged at least once in feeder-free conditions, on 0.1% gelatin-coated six-well plates and cultured in medium containing 40% CM+/+ and 60% BRL-conditioned CM+/+ medium. Expression of the Dam–POI in the mouse ES cell lines was suppressed by 0.5 mM indole-3-acetic acid (IAA; Sigma, I5148).

### Induction of Dam–POI expression

In KBM7 cells, Dam was induced with 0.5 nM Shield-1 (Glixx Laboratories Inc, GLXC-02939) that stabilizes the protein. In hybrid mouse ES cells, 24 h before induction the cells were cultured in 1 mM IAA (Sigma, I5148), to ensure protein degradation. For induction the cells were washed three times with PBS and fresh medium without IAA was added, followed by the addition of 1 μM 4-hydroxytamoxifen (Sigma, SML1666). In detail, Dam–LMNB1 mouse ES cells were induced for 6 h without IAA and Dam–scFv-H3K27me3 mouse ES cells were induced for 20 h without IAA and the last 6 h with 4-hydroxytamoxifen.

### In vitro differentiation of mouse hybrid ES cells with ascorbic acid (vitamin C)

ES cells were depleted from any remaining mouse embryonic fibroblasts and 24–48 h later confluent wells were passaged and plated in AA differentiation medium: IMDM supplemented with 15% FBS, 1% pen–strep, 1× GlutaMAX, 1× non-essential amino acids, 50 μg ml^−1^ ascorbic acid (Sigma, A4544) and 0.1 mM β-mercaptoethanol^58,59^. Expression of the Dam–POI was suppressed by addition of 0.5 mM IAA. The medium was refreshed every day during the course of differentiation.

### Cell-cycle synchronization with double thymidine block

KBM7 cells were passaged 48 h before the initiation of synchronizations and received 2 mM thymidine (Sigma, T1895) for 15 h, followed by 9 h of release and a second thymidine block for 13 h. Synchronization tests were performed before Dam&ChIC experiments, to define the cell-cycle progression of these cell lines based on the DNA content. In the single-cell Dam&ChIC experiment, KBM7 cells were synchronized with a double thymidine block as above. During the second thymidine block, 0.5 nM of Shield-1 was added to enable stabilization of Dam–LMNB1. Afterwards, both thymidine and Shield-1 were washed out, cells were released to progress through S-phase, and collected in G2 phase (8 h post-thymidine release; equivalent to 1 h in G2) and as early as possible in G1 phase (13 h post-thymidine release; equivalent to 1 h in G1). While the Dam-deposited ^m6^A mark is typically not propagated upon DNA replication in the absence of the Dam–POI, this setup ensures that (1) Dam–LMNB1 is still present during S and G2 at sufficient levels to allow recovery of LADs and (2) measurements of de novo ^m6^A from any non-degraded protein in G1 are prevented, because of insufficient time to accumulate DamID signal by that collection time point^35^.

### Preparation of samples for Dam&ChIC

#### Cell collection and permeabilization to isolate nuclei

Nuclei isolation was carried out as described previously^40^. In brief, cells were collected in 15-ml Falcon tubes and washed three times with room-temperature PBS0. After counting, 0.5–1 million cells per staining were used for permeabilization in ice-cold wash buffer 1 (20 mM HEPES, pH 7.5, 150 mM NaCl, 66.6 μg ml^−1^ spermidine (Sigma, S2626-1G), 1× cOmplete protease inhibitor cocktail (Roche, 11836170001), 0.05% saponin (Sigma, 47036-50G-F) and 2 mM EDTA), in protein low-binding tubes (Eppendorf, 0030108094). The samples were washed once at 300*g* for 4 min at 4 °C, before the addition of the primary antibody. For all subsequent steps, the nuclei were kept always on ice. Subsequent washes were carried out with the same centrifuge settings.

#### Cell collection, ethanol fixation and permeabilization

For ethanol fixation, cells were collected in 15-ml Falcon tubes and washed three times with room-temperature PBS0. After counting, 0.5–1 million cells were fixed with 70% ice-cold ethanol for 1 h at −20 °C. Fixed cells were washed once and permeabilized in ice-cold wash buffer 1F (20 mM HEPES, pH 7.5, 150 mM NaCl, 1× cOmplete protease inhibitor cocktail and 0.05% Tween). Afterwards, samples were stained with CellTrace dyes (CellTrace CFSE, CellTrace Far Red, CellTrace Yellow) for 20 min at 4 °C or frozen at −80 °C for long-term storage in wash buffer 1F that also contained 66.6 μg ml^−1^ spermidine, 2 mM EDTA and 10% dimethylsulfoxide (DMSO).

#### Sample multiplexing based on CellTrace staining

Samples stained with different combinations of CellTrace dyes were mixed in one ‘super-sample’ to enable parallel processing and minimize batch effects, as described before^39,40^. Specifically, samples from each day of the vitamin C differentiation time-course were stained with a unique combination of CellTrace dyes. This enabled the distinction of up to eight populations. In the synchronization and mitosis experiment, the G2 sample was stained with CellTrace Yellow and CellTrace CFSE, and the early G1 sample was stained with CellTrace CFSE, CellTrace Far Red and CellTrace Yellow (Extended Data Fig. 3b). For all experiments, equal cell numbers from each sample were combined together to a final total amount of 0.5–1 million cells for subsequent antibody staining.

### Antibody staining and pA-MNase targeting

Nuclei or fixed cells were stained with the primary antibody overnight at 4 °C on a roller, in wash buffer 1 or 1F, respectively. Specifically, anti-histone H3 (Abcam, ab176842) was used at 1:400 dilution, anti-LMNB1 (Abcam, ab16048) was used at 1:200 or 1:400 dilution; anti-H3K27me3 (Cell Signaling Technologies, 9733S) at 1:200 dilution; anti-H3K9me3 RM389 (Thermo Fisher, MA5-33395) at 1:200 dilution; anti-H3K4me3 (Thermo Fisher, MA5-11199) at 1:400 dilution; and anti-H2AK119Ub (Cell Signaling, D27C4) at 1:400 dilution. After one wash with wash buffer 2 (for nuclei; 20 mM HEPES, pH 7.5, 150 mM NaCl, 66.6 μg ml^−1^ spermidine, 1× cOmplete protease inhibitor cocktail and 0.05% saponin) or wash buffer 2F (for fixed cells; 20 mM HEPES, pH 7.5, 150 mM NaCl, 66.6 μg ml^−1^ spermidine, 1× cOmplete protease inhibitor cocktail and 0.05% Tween), pA-MNase was added at a final concentration of 3 ng μl^−1^ for KBM7 samples and 0.6 ng μl^−1^ for mouse ES cells and vitamin C samples, in the respective buffers. Hoechst 34580 was added in the same mix at a final concentration of 2.5 μg ml^−1^. The samples were incubated in a roller at 4 °C for 1 h, followed by two washes in the same wash buffer, and finally passed through a cell-strainer for sorting.

### Single-cell sorting and FACS gating strategies

FACS sorting was performed on a BD FACS Influx or BD FACS Jazz System, with sample and plate cooling. Gating was based on size (forward-side scatter), exclusion of doublets and Hoechst-defined DNA content (Extended Data Fig. 3b). For experiments in which different samples were multiplexed, an additional gating strategy based on fluorescence of CellTrace dyes ensured distinction of the different populations (Extended Data Fig. 3b). One cell per well was sorted in hard-shell 384-well plates that were pre-filled with 5 μl per well mineral oil using the Freedom EVO liquid handling platform (Tecan) and 100 nl per well wash buffer 3 (for nuclei; 20 mM HEPES, pH 7.5, 150 mM NaCl, 66.6 μg ml^−1^ spermidine and 0.05% Saponin) or wash buffer 3F (for fixed/permeabilized cells; 20 mM HEPES, pH 7.5, 150 mM NaCl, 66.6 μg ml^−1^ spermidine and 0.05% Tween). After sorting, plates were sealed with aluminium seals (Greinier, 676090), spun at 2,000*g* for 1–2 min at 4 °C, and kept at 4 °C until further processing.

### Single-cell Dam&ChIC

Liquid dispensions were performed using the Nanodrop II liquid handler (Innovadyne) and adaptor dispensions were performed using the Mosquito LV (STP Labtech), as described previously^27,30,33^. PA-MNase in single nuclei was activated by dispension of 100 nl per well of activation solution (wash buffer 3 or wash buffer 3F, containing 4 mM CaCl_2_). Plates were incubated at 4 °C for exactly 30 min, followed by dispension of 200 nl per well of stop solution (containing 0.04 M EGTA, 1.5% NP40 and 2 μg μl^−1^ Proteinase K). Plates were incubated at 65 °C for 6 h, followed by heat inactivation at 80 °C for 10 min. Plates were stored at −20 °C until further processing. After thawing plates on ice, 200 nl per well of Blunt-ending mix (containing 4 nl (0.02 U) Klenow, 4 nl (0.04 U) T4 PNK, 10 nl 10 mM dNTPs, 60 nl 10 mM ATP, 20 nl 25 mM MgCl_2_, 10 nl 50% PEG8000, 6 nl 20 μg μl^−1^ BSA, 60 nl 10× PNK buffer and 26 nl ultrapure water) were dispensed, followed by incubation at 37 °C for 30 min and heat inactivation at 75 °C for 20 min. Next, digestion with 400 nl per well DpnI mix (containing 20 nl (0.4 U) DpnI, 40 nl 10× PNK buffer, 10 nl 20 μg μl^−1^ BSA and 330 nl ultrapure water) was conducted, followed by incubation at 37 °C for 8 h and heat inactivation at 80 °C for 20 min. Finally, the content of each well was barcoded through dispension of 50 nl per well of DamID2 adaptors at a final concentration of 25 nM and dispension of 950 nl per well ligation mix (containing 75 nl (0.375 U) T4 ligase, 200 nl 10× ligase buffer and 675 nl ultrapure water) to a cumulative final volume of 2,000 nl per well, followed by incubation at 4 °C for 20 min, 16 °C for 16 h and heat inactivation at 65 °C for 10 min. After each dispension step, the plates were sealed with aluminium seals and spun at 2,000*g* for 1 min at 4 °C.

### Single-cell DamID-only and ChIC-only controls

For the DamID-only control, the single-cell Dam&ChIC protocol was performed as above, but the Blunt-ending mix did not contain the enzymes (Klenow and T4 PNK). Similarly, for the ChIC-only controls, the DpnI mix did not contain the DpnI enzyme.

### Bulk sortChIC

To generate a normalization control for the ChIC readout of Dam&ChIC data (Extended Data Fig. 1g), we performed sortChIC in a population of 10,000 sorted KBM7 nuclei, stained with an anti-histone H3 antibody. Similarly, for antibody titrations, we performed bulk sortChIC in populations of 100 sorted nuclei. This protocol is an adaptation of the original sortChIC protocol^30,40^, with omission of A-tailing and all reaction volumes upscaled to a bulk format: Nuclei were sorted in 0.5 ml low-binding tubes containing 5 μl of wash buffer 3/3 F, pA-MNase was activated with 5 μl respective activation solution and stopped with 10 μl Stop solution. For blunt ending, 10 μl of reaction mix (containing 0.2 μl Klenow, 0.2 μl T4 PNK, 0.5 μl 10 mM dNTPs, 3 μl 10 mM ATP, 1 μl 25 mM MgCl_2_, 0.5 μl 50% PEG8000, 0.3 μl 20 μg μl^−1^ BSA, 3 μl 10× PNK buffer and 1.3 μl ultrapure water) were added. Each sample was barcoded with the addition of 2 μl of a unique DamID2 adaptor^60^ at a final concentration of 25 nM, followed by addition of 48 μl ligation mix (containing 1 μl T4 ligase, 8 μl 10× ligase buffer and 39 μl ultrapure water), to a cumulative volume of 80 μl. All incubations were carried out as described in the single-cell Dam&ChIC protocol.

### Single-cell Dam&T-seq

Single-cell Dam&T-seq was performed as described previously^27,29,33^ with major reductions in reaction volumes, to facilitate cost efficiency. One live cell per well was sorted in hard-shell 384-well plates that were pre-filled with 5 μl per well mineral oil and 50 nl per well of 1.5 μM barcoded CELseq2 primers^29,33^. After sorting, plates were sealed with aluminium seals, spun at 2,000*g* for 1–2 min at 4 °C, and frozen at −80 °C until further processing. In brief, the volumes per step were adapted to 50 nl per well of Lysis mix, 75 nl per well of RT mix, 925 nl per well of Second Strand Synthesis mix, 250 nl per well of Proteinase K mix and 150 nl per well of DpnI mix. Finally, 100 nl per well of barcoded DamID2 adaptors^33^ were added to a final concentration of 25 nM and ligated with the addition of 400 nl per well of ligation mix to a cumulative final volume of 2,000 nl per well.

### Library preparation

The content of all wells in a plate was pooled by centrifugation of the 384-well plates on collection plates, transferred to Eppendorf tubes, and the aqueous phase was separated from the oil phase by a few rounds of centrifugation and transfers to clean tubes. Sample purification with beads followed, as described previously^78^, by incubation of the sample with 0.8 volumes of 1:10 diluted beads (CleanNGS; GC Biotech, CNGS-0050) in bead-binding buffer (1 M NaCl, 20% PEG8000, 20 mM Tris-HCl, pH 8 and 1 mM EDTA), three washes with 80% ethanol and elusion with 7 μl ultrapure nuclease-free water. The eluted sample was then in vitro transcribed at 37 °C for 14 h, using the MEGAScript T7 transcription kit (Invitrogen, AMB13345). For single-cell Dam&ChIC, the amplified RNA (aRNA) was purified using 0.8 volume beads, washed three times with 80% ethanol and eluted in 13 μl ultrapure nuclease-free water. The quality of the eluate was evaluated using the Agilent RNA 6000 Pico Assay and, if necessary, it was bead-purified again. For scDam&T-seq, upon first purification, the aRNA was fragmented with the addition of 0.2 volume fragmentation buffer (500 mM potassium acetate, 150 mM magnesium acetate and 200 mM Tris acetate) at 94 °C for 90 s, and the reaction was stopped on ice and quenched with 0.1 volume 0.5 M EDTA, followed by another 0.8 volume bead cleanup. A maximum of 6 μl of 100–200 ng aRNA was used for reverse transcription, followed by 8–11 cycles of library PCR, depending on the amount of input aRNA, and the amplified material was purified twice, as described above, before final elution in 13 μl of ultrapure nuclease-free water. Libraries were quantified using the Qubit dsDNA High Sensitivity Assay and the Agilent High Sensitivity DNA Assay, and subsequently sequenced with single-end or paired-end sequencing on the Illumina NextSeq500 (75-bp reads) or the Illumina NextSeq2000 (100-bp reads).

### Oligonucleotides

Oligonucleotides used for Dam&ChIC have been described previously^33^. DamID2 adaptor sequences can be found in Supplementary Table 1, as top and bottom oligonucleotides used for annealing. Random hexamer and library PCR primers can be found in Supplementary Table 2.

### Data processing

#### Dam&ChIC data processing

Dam&ChIC data processing was largely based on the workflow and scripts described elsewhere^29,33^, but adapted to allow for computational separation of DamID and ChIC-derived reads and further processing of ChIC reads. The key steps are described below (explanation and code are available on GitHub at https://github.com/KindLab/DamChIC).

##### Raw data pre-processing

Reads were demultiplexed by comparing their sample barcode at the start of each read (R1 in case of paired-end sequenced libraries) conforming to the read layout of 5′-(3 nt UMI)(8 nt barcode)TC(gDNA)−3′ for DamID and 5′-(3 nt UMI)(8 nt barcode)WN(gDNA)-3′ for ChIC reads, to a reference list of barcodes and zero mismatches between the observed barcode and reference are allowed. Sample barcodes and UMIs were trimmed off the reads to retain only gDNA sequences. The UMI sequences were appended to the read name for downstream processing. Per sample barcode, DamID and ChIC reads remained mixed at this point.

##### Sequence alignments

The reads were aligned to a reference genome using bowtie2 (v.2.4.1) with parameters ‘--seed 42 --very-sensitive -N 1’. Before alignment, a ‘GA’ dinucleotide was prepended to the reads that was digested off DamID fragments during DpnI gDNA digestion. For human samples, reads were aligned to the reference genome hg19 (GRCh37) and mouse samples to mm10 (GRCm38). For allele-specific processing for XCI experiments, data were aligned to reference genomes generated by imputing 129/Sv and CAST/EiJ single-nucleotide polymorphisms obtained from the Sanger Mouse Genomes project^72^ onto the mm10 reference genome. The reads were assigned to either genotype by aligning reads to both references. Reads that aligned with lower edit distance (SAM tag ‘NM’) or higher alignment score (SAM tag ‘AS’) in case of equal edit distance to one of the genotypes were assigned to that genotype. Reads aligning with equal edit distance and alignment score to both genotypes were considered of ‘ambiguous’ genotype. Reads that yielded an alignment with mapping quality (BAM field ‘MAPQ’) lower than 10 were discarded.

##### In silico separation of DamID and ChIC reads

Reads were considered DamID in case of perfect alignment on genomic GATC motifs. Residual reads were considered ChIC and their original sequences (that is, without prepended GA dinucleotide) were realigned using hisat2 (v.2.1.0) with parameters ‘--seed 42 --no-spliced-alignment --mp 2,0 --sp 4,0’. These ChIC-considered reads were further pruned given MNase’s strong adenine or thymidine-nucleotide cut preference (Extended Data Fig. 1c); only ChIC reads were kept that start with an ‘A’ or ‘T’ nucleotide. In addition, we observed that a larger fraction of ChIC reads (those that do not map on a genomic GATC motif) are ‘TC’-dinucleotide starting in Dam&ChIC libraries than in ChIC libraries (Extended Data Fig. 1d) and excluded TC-starting reads from Dam&ChIC-derived ChIC libraries.

##### PCR duplicate filtering

To exclude PCR read duplicates, both DamID and ChIC read counts were collapsed based on genomic position, strand and UMI sequence. In case of haploid KBM7 cells or allele-specific data processing, multiple reads with the same UMI counted as 1 and for diploid data processing, as 2. The number of observed unique UMIs was taken as the number of unique methylation events for DamID or unique MNase cut sites for ChIC.

##### Filtering of samples

To exclude single-cell samples from the analysis that failed we applied construct-specific cutoffs on UMI-unique reads for DamID: 1,000 for Dam and Dam–scFv-H3K27me3 and 5,000 for Dam–LMNB1. A general cutoff of 1,000 UMI-unique reads per sample was applied for ChIC. Samples in allele-specific analysis were included if ≥200 UMI-unique reads could be assigned to both parental alleles for DamID as well as ChIC-derived reads.

#### scDam&T-seq data processing

The scDam&T-seq data were processed as before (explanation and code available on GitHub at https://github.com/KindLab/scDamAndTools)^33^. For allele-specific processing, data were aligned to reference genomes generated by imputing 129/Sv and CAST/EiJ single-nucleotide polymorphisms obtained from the Sanger Mouse Genomes project^79^ onto the mm10 reference genome. The reads were assigned to either genotype by aligning reads to both references. Reads that aligned with lower edit distance (SAM tag ‘NM’) or higher alignment score (SAM tag ‘AS’) in case of equal edit distance to one of the genotypes were assigned to that genotype. Reads aligning with equal edit distance and alignment score to both genotypes were considered of ‘ambiguous’ genotype. Reads that yielded an alignment with mapping quality (BAM field ‘MAPQ’) lower than 10 were discarded.

##### Filtering of samples

Single-cell samples for allele-specific analysis were included if ≥200 UMI-unique reads could be assigned to both parental alleles for DamID and ≥200 UMI-unique transcripts.

#### ChIP-seq data processing

External K562 ChIP-seq datasets were downloaded from the ENCODE database^80^. ChIP-seq reads were aligned using bowtie2 (v.2.4.1) with parameters ‘--seed 42 --very-sensitive -N 1’. ChIP-seq data were binned using the ‘bamCoverage’ function from DeepTools (v.3.3.2) with parameters ‘--ignoreDuplicates --minMappingQuality 10’. NarrowPeak calls were likewise downloaded from the ENCODE database from the corresponding ChIP-seq datasets.

### Downstream analyses

#### Binning and calculation of OE values

DamID and ChIC data were binned using consecutive non-overlapping 100-kb bins. For gene-based analysis (Fig. 1c,d) data were binned at a higher resolution of 1 kb. To calculate OE values^7,29^, an ‘expected’ vector for DamID data was generated in silico by generating sequences of 65 nt (in both orientations) from the reference genome and aligning and processing them identically to the data. By binning the in silico-generated reads, the maximum amount of mappable unique events per bin was determined (Extended Data Fig. 1g, top). The expected for ChIC was generated experimentally by targeting pA-MNase to histone H3 (Extended Data Fig. 1g, bottom), unless stated otherwise.

#### Data binarization, contact frequency and combined single-cell heatmap

If specifically stated, data were binarized by setting a threshold of log_2_(≥1) based on the bimodal distribution of OE values of 100-kb genomic bins (Extended Data Fig. 2a)^7^. The CF metric is used throughout the manuscript and is defined by calculating the fraction of single cells that meet the binarization threshold for a given 100-kb bin^7^. The combined single-cell heatmap presented in Fig. 3c is determined by first binarizing the 100-kb binned data of both Dam&ChIC measurements separately, resulting in two presence–absence m × n matrices; (1) DamID defined as Md = aij and (2) ChIC defined Mc = aij, where i is a cell and j a 100-kb bin. Both matrices are combined in one by simple summation as (Md + Mc) + Mc. In the resulting combined matrix 0 denotes absence of both, 1 denotes the presence of only DamID, 2 denotes the presence of only ChIC and 3 denotes the presence of both.

#### Dimensionality reduction on KBM7 Dam&ChIC data

The UMAP presented in Fig. 1f was computed by first calculating the first 50 principal components (Python; sklearn v.1.0.2) on the RPKM normalized single-cell data binned at 100-kb resolution, on which UMAP (Python; umap v.0.5.2) was then run.

#### Single-cell LAD calling

Bulk LADs presented in Extended Data Fig. 1c are called by setting a threshold of log_2_(≥1) based on the bimodal distribution of OE values of 100-kb genomic bins Extended Data Fig. 1a^7^. Notably, LAD calls <300 kb were excluded and LAD calls intervened by ≤100 kb were combined into one. For single-cell LAD calling used for Fig. 2, LADs we first separately called on pseudobulk profiles of both Dam&ChIC readouts as described above. Only LADs that were identified by both readouts were kept for single-cell analysis (Extended Data Fig. 2c). The resulting LAD co-ordinates were in each cell tested for having an average OE log_2_(≥1), if this requirement was met, the LAD was called in the given cell. This resulted in two LAD-based binary presence–absence matrices (1) DamID defined as Md = aij and (2) ChIC defined as Mc = aij, where i represents a cell and j represents a LAD.

#### Jaccard *z*-score normalization by matrix permutation

To compare the single-cell co-occurrence of DamID- and ChIC-identified LADs between different Dam&ChIC experiments and across LADs with different CFs, the Jaccard similarity index was computed, defined as JA,B = A⋂BA⋃B, where A represents the DamID and B the ChIC measurement from the same cell (Fig. 3b and Extended Data Figs. 2f and 3e). Two inherent problems are posed on this analysis that might introduce undesirable bias and ultimately unreliable comparison between different experiments or LADs: (1) typical sparsity of single-cell data results in non-uniformly distributed signal dropout and (2) binary similarity metrics can be sensitive to site prominence that varies between different LADs, but which does not reflect true co-occurrence of DamID and ChIC signal. To solve both problems, we applied a previously described matrix randomization algorithm^81^, to permute the aforementioned presence–absence LAD matrix Mc *n* times (*n* = 100), without altering row and column totals. The resulting randomized matrices were used to compute Jaccard similarity with LAD matrix Md to serve for *z*-score normalization, which can be written as *z* = *x* − μ, where *x* is the similarity score of the observed, the mean similarity and the s.d. of the random controls.

#### Chromatin velocity

Chromatin velocity analysis presented in Figs. 2 and 6 were performed using the scVelo Python package (v.0.2.5)^37^. An average OE value was calculated in the abovementioned LAD calls for each cell for both the Dam–LMNB1 and LMNB1 Dam&ChIC readout. The resulting Dam–LMNB1 matrix was provided to scVelo as the ‘spliced’ layer and the LMNB1 matrix as ‘unspliced’.

#### XCI dimensionality reduction and trajectory inference

Dimensionality reduction of the XCI analysis was performed with Seurat (v.4.1.0)^82^ and Signac (v.1.3.0)^83^. The UMAP introduced in Fig. 5b is based on H3K27me3 signal on autosomal genes (5 kb upstream of TSS + gene body). Different batches (Dam&ChIC experiments) were integrated using Harmony (v.0.1.0)^84^. Pseudotime inference was performed using Monole3 (v.1.0.0)^68^.

#### *z*-score normalized cell cycle analysis on XCI data

The cell-cycle analysis on the XCI dataset presented in Fig. 6 is based on Hoechst staining of sorted cells, which reflects DNA content and therefore enables their categorization to the different cell cycle phases (G1, S or G2). However, these phases are not equally divided across single cells, as the majority of the cells in culture are in G1 phase (Extended Data Fig. 6e,g). To correct for this, we performed *z*-score normalization of the observed cell cycle distributions within *k*-nearest neighbours (*k*-NN; *k* = 100). For each neighbourhood, the observed cell cycle distribution was compared with *n*-times randomly sampled controls (*n* = 100). The *z*-score normalization was defined as follows, *z* = *x* − μ, where *x* is the cell cycle distribution of the observed, the mean similarity and the s.d. of the randomly sampled controls. The resulting score for each cell represents the cell cycle phase enrichment for the neighbourhood of that cell.

#### Definition of start- and end-point anchors in latent time ordering

For the analysis in Fig. 2h, the start and end point of LADs through latent time were determined as follows. All bins of each LAD were averaged over latent time, resulting in a one-dimensional vector containing LAD intensity as a function of latent time. These vectors were first smoothed using a Gaussian filter (sigma = 5) before the start increase and end decay were determined using a binary segmentation algorithm ruptures.Binseg^85^. From these single start and end points, 75 cells upstream and 10 cells downstream in latent time were averaged.

#### Simulating in silico data

To corroborate our interpretation of the temporal relationship between chromatin features in Fig. 5, in silico data were simulated and presented in Extended Data Fig. 5a–c. The in silico data were generated by an adapted function from scvelo.datasets.simulation^37^. In brief, the parameters for each reaction were randomly sampled from a log-normal distribution, and time events followed the Poisson law. Both the switch moment and noise levels were controlled. The adapted function is made available on GitHub at https://github.com/KindLab/DamChIC.

#### Definition of the inactive X chromosome

The Xi chromosome (Cast/EiJ or 129/Sv allele) was determined based on one of the two measured features. In the case of Dam–LMNB1/H3K27me3 Dam&ChIC data the allele with the highest H3K27me3 signal was defined as the Xi. For Dam–LMNB1/H2AK119Ub Dam&ChIC, this was the allele with the highest H2AK119Ub signal. For both Dam–LMNB1/H3K9me3 and Dam–LMNB1/LMNB1 Dam&ChIC data, this was the allele with the lowest Dam–LMNB1 signal. For all Dam&ChIC datasets that involved the Dam–scFv-H3K27me3 DamID construct, the allele with the highest levels of H3K27me3 DamID signal was selected. Finally, for the scDam&T-seq dataset in Dam–LMNB1-expressing cells, the RNA expression levels of Xist were used.

### Statistics and reproducibility

No statistical method was used to predetermine sample size. Cells of low quality were excluded as described in Methods. The experiments were not randomized and the investigators were not blinded to allocation during experiments and outcome assessment. Each plate contained cells of all experimental conditions to be compared, to ensure identical processing and minimize batch effects.

### Reporting summary

Further information on research design is available in the Nature Portfolio Reporting Summary linked to this article.

## Online content

Any methods, additional references, Nature Portfolio reporting summaries, source data, extended data, supplementary information, acknowledgements, peer review information; details of author contributions and competing interests; and statements of data and code availability are available at 10.1038/s41556-025-01687-w.

## Supplementary information

Reporting Summary

Supplementary Tables DamID2 adaptor sequences and oligonucleotides for library preparation. Hoechst DNA staining values along with cell cycle phase calls related to Extended Data Fig. 6.

## Source data

Source Data Figs. 1–6 and Extended Data Figs. 1–6 Numerical data underlying plots.

## Data Availability

All genomic and transcriptomic data generated in this study have been deposited at the Gene Expression Omnibus (GEO) under accession number GSE247458 and GSE288852. Previously published K562 ChIP-seq data that were used to benchmark Dam&ChIC data are available in the ENCODE database with numbers ENCSR668LDD (H3K4me3), ENCSR000EWB (H3K27me3) and ENCSR000APE (H3K9me3). Likewise, K562 ATAC-seq data are available in the ENCODE database (ENCSR956DNB). The data of previously published single-cell multifactorial profiling methods that were used to compare to Dam&ChIC have been deposited under GEO under accession numbers GSE171554 (multi-CUT&Tag;^12^), GSE179756 (MulTI-Tag;^13^), GSE212588 (NTT-seq;^15^) and GSE198467 (Nano-CT;^14^). Alu/SINE and L1/LINE annotations (Extended Data Fig. 3) were obtained from the RepeatMasker database (repeatmasker.org). Previously published CHART-seq data^56^ that were re-analysed here are available under GEO accession number GSE48649. Source data are provided with this paper.

## Code availability

All custom code generated as part of this study have been deposited on GitHub at https://github.com/KindLab/DamChIC.

## References

[1] Millán-Zambrano, G., Burton, A., Bannister, A. J. & Schneider, R. Histone post-translational modifications — cause and consequence of genome function. *Nat. Rev. Genet.* **23**, 563–580 (2022). 35338361 10.1038/s41576-022-00468-7

[2] van Steensel, B. & Furlong, E. E. M. The role of transcription in shaping the spatial organization of the genome. *Nat. Rev. Mol. Cell Biol.* **20**, 327–337 (2019). 30886333 10.1038/s41580-019-0114-6 PMC7116054

[3] van Steensel, B. & Belmont, A. S. Lamina-associated domains: links with chromosome architecture, heterochromatin, and gene repression. *Cell* **169**, 780–791 (2017). 28525751 10.1016/j.cell.2017.04.022 PMC5532494

[4] Nagano, T. et al. Single-cell Hi-C reveals cell-to-cell variability in chromosome structure. *Nature* **502**, 59–64 (2013). 24067610 10.1038/nature12593 PMC3869051

[5] Buenrostro, J. D. et al. Single-cell chromatin accessibility reveals principles of regulatory variation. *Nature* **523**, 486–490 (2015). 26083756 10.1038/nature14590 PMC4685948

[6] Cusanovich, D. A. et al. Multiplex single-cell profiling of chromatin accessibility by combinatorial cellular indexing. *Science* **348**, 910–914 (2015). 25953818 10.1126/science.aab1601 PMC4836442

[7] Kind, J. et al. Genome-wide maps of nuclear lamina interactions in single human cells. *Cell* **163**, 134–147 (2015). 26365489 10.1016/j.cell.2015.08.040 PMC4583798

[8] Rotem, A. et al. Single-cell ChIP-seq reveals cell subpopulations defined by chromatin state. *Nat. Biotechnol.* **33**, 1165–1172 (2015). 26458175 10.1038/nbt.3383 PMC4636926

[9] Jin, W. et al. Genome-wide detection of DNase I hypersensitive sites in single cells and FFPE tissue samples. *Nature* **528**, 142–146 (2015). 26605532 10.1038/nature15740 PMC4697938

[10] Lai, B. et al. Principles of nucleosome organization revealed by single-cell micrococcal nuclease sequencing. *Nature* **562**, 281–285 (2018). 30258225 10.1038/s41586-018-0567-3 PMC8353605

[11] Grosselin, K. et al. High-throughput single-cell ChIP-seq identifies heterogeneity of chromatin states in breast cancer. *Nat. Genet.* **51**, 1060–1066 (2019). 31152164 10.1038/s41588-019-0424-9

[12] Ku, W. L. et al. Single-cell chromatin immunocleavage sequencing (scChIC-seq) to profile histone modification. *Nat. Methods* **16**, 323–325 (2019). 30923384 10.1038/s41592-019-0361-7 PMC7187538

[13] Carter, B. et al. Mapping histone modifications in low cell number and single cells using antibody-guided chromatin tagmentation (ACT-seq). *Nat. Commun.* **10**, 3747 (2019). 31431618 10.1038/s41467-019-11559-1 PMC6702168

[14] Kaya-Okur, H. S. et al. CUT&Tag for efficient epigenomic profiling of small samples and single cells. *Nat. Commun.* **10**, 1930 (2019). 31036827 10.1038/s41467-019-09982-5 PMC6488672

[15] Bartosovic, M., Kabbe, M. & Castelo-Branco, G. Single-cell CUT&Tag profiles histone modifications and transcription factors in complex tissues. *Nat. Biotechnol.* **39**, 825–835 (2021). 33846645 10.1038/s41587-021-00869-9 PMC7611252

[16] Wang, Q. et al. CoBATCH for high-throughput single-cell epigenomic profiling. *Mol. Cell* **76**, 206–216.e7 (2019). 31471188 10.1016/j.molcel.2019.07.015

[17] Wu, S. J. et al. Single-cell CUT&Tag analysis of chromatin modifications in differentiation and tumor progression. *Nat. Biotechnol.* **39**, 819–824 (2021). 33846646 10.1038/s41587-021-00865-z PMC8277750

[18] Arrastia, M. V. et al. Single-cell measurement of higher-order 3D genome organization with scSPRITE. *Nat. Biotechnol.* **40**, 64–73 (2022). 34426703 10.1038/s41587-021-00998-1 PMC11588347

[19] Gopalan, S., Wang, Y., Harper, N. W., Garber, M. & Fazzio, T. G. Simultaneous profiling of multiple chromatin proteins in the same cells. *Mol. Cell* **81**, 4736–4746 e5 (2021). 34637755 10.1016/j.molcel.2021.09.019 PMC8604773

[20] Meers, M. P., Llagas, G., Janssens, D. H., Codomo, C. A. & Henikoff, S. Multifactorial profiling of epigenetic landscapes at single-cell resolution using MulTI-Tag. *Nat. Biotechnol.* **41**, 708–716 (2023). 36316484 10.1038/s41587-022-01522-9 PMC10188359

[21] Bartosovic, M. & Castelo-Branco, G. Multimodal chromatin profiling using nanobody-based single-cell CUT&Tag. *Nat. Biotechnol.* **41**, 794–805 (2023). 36536148 10.1038/s41587-022-01535-4 PMC10264246

[22] Stuart, T. et al. Nanobody-tethered transposition enables multifactorial chromatin profiling at single-cell resolution. *Nat. Biotechnol.* **41**, 806–812 (2023). 36536150 10.1038/s41587-022-01588-5 PMC10272075

[23] Lochs, S. J. A. et al. Combinatorial single-cell profiling of all major chromatin types with MAbID. *Nat. Methods* **21**, 72–82 (2024). 38049699 10.1038/s41592-023-02090-9 PMC10776404

[24] van Steensel, B. & Henikoff, S. Identification of in vivo DNA targets of chromatin proteins using tethered dam methyltransferase. *Nat. Biotechnol.* **18**, 424–428 (2000). 10748524 10.1038/74487

[25] Vogel, M. J., Peric-Hupkes, D. & van Steensel, B. Detection of in vivo protein-DNA interactions using DamID in mammalian cells. *Nat. Protoc.***2**, 1467–1478 (2007).17545983 10.1038/nprot.2007.148

[26] Guelen, L. et al. Domain organization of human chromosomes revealed by mapping of nuclear lamina interactions. *Nature* **453**, 948–951 (2008). 18463634 10.1038/nature06947

[27] Rang, F. J. et al. Single-cell profiling of transcriptome and histone modifications with EpiDamID. *Mol. Cell* **82**, 1956–1970 e14 (2022). 35366395 10.1016/j.molcel.2022.03.009 PMC9153956

[28] Boers, R. et al. Retrospective analysis of enhancer activity and transcriptome history. *Nat. Biotechnol.* **41**, 1582–1592 (2023). 36823354 10.1038/s41587-023-01683-1 PMC10635829

[29] Rooijers, K. et al. Simultaneous quantification of protein-DNA contacts and transcriptomes in single cells. *Nat. Biotechnol.* **37**, 766–772 (2019). 31209373 10.1038/s41587-019-0150-y PMC6609448

[30] Zeller, P. et al. Single-cell sortChIC identifies hierarchical chromatin dynamics during hematopoiesis. *Nat. Genet.* **55**, 333–345 (2023). 36539617 10.1038/s41588-022-01260-3 PMC9925381

[31] Schmid, M., Durussel, T. & Laemmli, U. K. ChIC and ChEC; genomic mapping of chromatin proteins. *Mol. Cell* **16**, 147–157 (2004). 15469830 10.1016/j.molcel.2004.09.007

[32] Skene, P. J. & Henikoff, S. An efficient targeted nuclease strategy for high-resolution mapping of DNA binding sites. *eLife* **6**, e21856 (2017). 28079019 10.7554/eLife.21856 PMC5310842

[33] Markodimitraki, C. M. et al. Simultaneous quantification of protein-DNA interactions and transcriptomes in single cells with scDam&T-seq. *Nat. Protoc.* **15**, 1922–1953 (2020). 32350457 10.1038/s41596-020-0314-8 PMC7779467

[34] Dingwall, C., Lomonossoff, G. P. & Laskey, R. A. High sequence specificity of micrococcal nuclease. *Nucleic Acids Res.* **9**, 2659–2673 (1981). 6269057 10.1093/nar/9.12.2659 PMC326883

[35] Kind, J. et al. Single-cell dynamics of genome-nuclear lamina interactions. *Cell* **153**, 178–192 (2013). 23523135 10.1016/j.cell.2013.02.028

[36] La Manno, G. et al. RNA velocity of single cells. *Nature* **560**, 494–498 (2018). 30089906 10.1038/s41586-018-0414-6 PMC6130801

[37] Bergen, V., Lange, M., Peidli, S., Wolf, F. A. & Theis, F. J. Generalizing RNA velocity to transient cell states through dynamical modeling. *Nat. Biotechnol.* **38**, 1408–1414 (2020). 32747759 10.1038/s41587-020-0591-3

[38] Tedesco, M. et al. Chromatin velocity reveals epigenetic dynamics by single-cell profiling of heterochromatin and euchromatin. *Nat. Biotechnol.* **40**, 235–244 (2022). 34635836 10.1038/s41587-021-01031-1

[39] Yeung, J. et al. scChIX-seq infers dynamic relationships between histone modifications in single cells. *Nat. Biotechnol.* **41**, 813–823 (2023). 36593403 10.1038/s41587-022-01560-3 PMC10264247

[40] Gaza, H. V., Bhardwaj, V. & Zeller, P. in *Chromatin Immunoprecipitation* (ed. Greulich, F.) vol. 2846 215–241 (Springer US, 2024). 10.1007/978-1-0716-4071-5_14 39141239

[41] LYON, M. F. Gene action in the X-chromosome of the mouse (*Mus musculus* L.). *Nature* **190**, 372–373 (1961). 13764598 10.1038/190372a0

[42] Lyon, M. F. Sex chromatin and gene action in the mammalian X-chromosome. *Am. J. Hum. Genet.* **14**, 135–148 (1962). 14467629 PMC1932279

[43] Heard, E., Chaumeil, J., Masui, O. & Okamoto, I. Mammalian X-chromosome inactivation: an epigenetics paradigm. *Cold Spring Harb. Symposia Quant. Biol.* **69**, 89–102 (2004). 16117637 10.1101/sqb.2004.69.89

[44] Brown, C. J. et al. The human X/ST gene: analysis of a 17 Kb inactive X-specific RNA that contains conserved repeats and is highly localized within the nucleus. *Cell* **71**, 527–642 (1992). 1423611 10.1016/0092-8674(92)90520-m

[45] Panning, B. & Dausman, J. X Chromosome inactivation is mediated by Xist RNA stabilization. *Cell* **90**, 907–916 (1997). 9298902 10.1016/s0092-8674(00)80355-4

[46] Penny, G. D., Kay, G. F., Sheardown, S. A., Rastan, S. & Neil Brockdorfft, B. Requirement for Xist in X chromosome inactivation. *Nature* **379**, 131–137 (1996). 8538762 10.1038/379131a0

[47] Plath, K. et al. Role of histone H3 lysine 27 methylation in X inactivation. *Science* **300**, 131–135 (2003). 12649488 10.1126/science.1084274

[48] Silva, J. et al. Establishment of histone H3 methylation on the inactive X chromosome requires transient recruitment of Eed-Enx1 polycomb group complexes. *Dev. Cell* **4**, 481–495 (2003). 12689588 10.1016/s1534-5807(03)00068-6

[49] De Napoles, M. et al. Polycomb group proteins Ring1A/B link ubiquitylation of histone H2A to heritable gene silencing and X inactivation. *Dev. Cell* **7**, 663–676 (2004). 15525528 10.1016/j.devcel.2004.10.005

[50] Żylicz, J. J. et al. The implication of early chromatin changes in X chromosome inactivation. *Cell* **176**, 182–197.e23 (2019). 30595450 10.1016/j.cell.2018.11.041 PMC6333919

[51] Belmont, A. S., Bignone, F. & Ts’O, P. O. P. The relative intranuclear positions of barr bodies in XXX non-transformed human fibroblasts. *Exp. Cell. Res.* **165**, 165–179 (1986). 3709685 10.1016/0014-4827(86)90541-0

[52] Bourgeois, C. A., Laquerriere, F., Hemon, D., Hubert, J. & Bouteille, M. New data on the in situ position of the inactive X chromosome in the interphase nucleus of human fibroblasts. *Hum. Genet* **69**, 122–129 (1985). 3972413 10.1007/BF00293281

[53] Zhang, L. F., Huynh, K. D. & Lee, J. T. Perinucleolar targeting of the inactive X during S phase: evidence for a role in the maintenance of silencing. *Cell* **129**, 693–706 (2007). 17512404 10.1016/j.cell.2007.03.036

[54] Rego, A., Sinclair, P. B., Tao, W., Kireev, I. & Belmont, A. S. The facultative heterochromatin of the inactive X chromosome has a distinctive condensed ultrastructure. *J. Cell Sci.* **121**, 1119–1127 (2008). 18334550 10.1242/jcs.026104

[55] Teller, K. et al. A top-down analysis of Xa- and Xi-territories reveals differences of higher order structure at ≥20 Mb genomic length scales. *Nucleus* **2**, 465–477 (2011). 21970989 10.4161/nucl.2.5.17862

[56] Augui, S., Nora, E. P. & Heard, E. Regulation of X-chromosome inactivation by the X-inactivation centre. *Nat. Rev. Genet.* **12**, 429–442 (2011). 21587299 10.1038/nrg2987

[57] Chen, C. K. et al. Xist recruits the X chromosome to the nuclear lamina to enable chromosome-wide silencing. *Science* **354**, 468–472 (2016). 27492478 10.1126/science.aae0047

[58] Loda, A. et al. Genetic and epigenetic features direct differential efficiency of Xist-mediated silencing at X-chromosomal and autosomal locations. *Nat. Commun.* **8**, 690 (2017). 28947736 10.1038/s41467-017-00528-1 PMC5612955

[59] Robert-Finestra, T. et al. SPEN is required for Xist upregulation during initiation of X chromosome inactivation. *Nat. Commun.* **12**, 7000 (2021). 34853312 10.1038/s41467-021-27294-5 PMC8636516

[60] Guerreiro, I. et al. Antagonism between H3K27me3 and genome–lamina association drives atypical spatial genome organization in the totipotent embryo. *Nat. Genet.* **56**, 2228–2237 (2024). 39284976 10.1038/s41588-024-01902-8 PMC11525175

[61] Schoeftner, S. et al. Recruitment of PRC1 function at the initiation of X inactivation independent of PRC2 and silencing. *EMBO J.* **25**, 3110–3122 (2006). 16763550 10.1038/sj.emboj.7601187 PMC1500994

[62] Tavares, L. et al. RYBP-PRC1 complexes mediate H2A ubiquitylation at polycomb target sites independently of PRC2 and H3K27me3. *Cell* **148**, 664–678 (2012). 22325148 10.1016/j.cell.2011.12.029 PMC3281992

[63] Almeida, M. et al. PCGF3/5–PRC1 initiates Polycomb recruitment in X chromosome inactivation. *Science* **356**, 1081–1084 (2017). 28596365 10.1126/science.aal2512 PMC6522364

[64] Vallot, C. et al. Erosion of X chromosome inactivation in human pluripotent cells initiates with XACT coating and depends on a specific heterochromatin landscape. *Cell Stem Cell* **16**, 533–546 (2015). 25921272 10.1016/j.stem.2015.03.016

[65] Keniry, A. et al. Setdb1-mediated H3K9 methylation is enriched on the inactive X and plays a role in its epigenetic silencing. *Epigenetics Chromatin* **9**, 16 (2016). 27195021 10.1186/s13072-016-0064-6 PMC4870784

[66] Simon, M. D. et al. High-resolution Xist binding maps reveal two-step spreading during X-chromosome inactivation. *Nature* **504**, 465–469 (2013). 24162848 10.1038/nature12719 PMC3904790

[67] Trapnell, C. et al. The dynamics and regulators of cell fate decisions are revealed by pseudotemporal ordering of single cells. *Nat. Biotechnol.* **32**, 381–386 (2014). 24658644 10.1038/nbt.2859 PMC4122333

[68] Cao, J. et al. The single-cell transcriptional landscape of mammalian organogenesis. *Nature* **566**, 496–502 (2019). 30787437 10.1038/s41586-019-0969-x PMC6434952

[69] Oksuz, O. et al. Capturing the onset of PRC2-mediated repressive domain formation. *Mol. Cell* **70**, 1149–1162.e5 (2018). 29932905 10.1016/j.molcel.2018.05.023 PMC7700016

[70] Zhao, J., Sun, B. K., Erwin, J. A., Song, J.-J. & Lee, J. T. Polycomb proteins targeted by a short repeat RNA to the mouse X chromosome. *Science* **322**, 750–756 (2008). 18974356 10.1126/science.1163045 PMC2748911

[71] Chu, C. et al. Systematic discovery of Xist RNA binding proteins. *Cell* **161**, 404–416 (2015). 25843628 10.1016/j.cell.2015.03.025 PMC4425988

[72] De Luca, K. L. et al. Genome-wide profiling of DNA repair proteins in single cells. *Nat. Commun.* **15**, 9918 (2024). 39572529 10.1038/s41467-024-54159-4 PMC11582664

[73] Horton, J. R., Liebert, K., Hattman, S., Jeltsch, A. & Cheng, X. Transition from nonspecific to specific DNA interactions along the substrate-recognition pathway of Dam methyltransferase. *Cell* **121**, 349–361 (2005). 15882618 10.1016/j.cell.2005.02.021 PMC2656680

[74] Pollex, T. & Heard, E. Nuclear positioning and pairing of X-chromosome inactivation centers are not primary determinants during initiation of random X-inactivation. *Nat. Genet.* **51**, 285–295 (2019). 30643252 10.1038/s41588-018-0305-7 PMC7617203

[75] Siegenfeld, A. P. et al. Polycomb-lamina antagonism partitions heterochromatin at the nuclear periphery. *Nat. Commun.* **13**, 4199 (2022). 35859152 10.1038/s41467-022-31857-5 PMC9300685

[76] Colognori, D., Sunwoo, H., Kriz, A. J., Wang, C.-Y. & Lee, J. T. Xist deletional analysis reveals an interdependency between Xist RNA and polycomb complexes for spreading along the inactive X. *Mol. Cell* **74**, 101–117.e10 (2019). 30827740 10.1016/j.molcel.2019.01.015 PMC6469964

[77] Kefalopoulou, S. & Zeller, P. Dam&ChIC. *protocols.io*10.17504/protocols.io.6qpvrkdbplmk/v1 (2025).

[78] Hashimshony, T. et al. CEL-Seq2: sensitive highly-multiplexed single-cell RNA-seq. *Genome Biol.* **17**, 77 (2016). 27121950 10.1186/s13059-016-0938-8 PMC4848782

[79] Keane, T. M. et al. Mouse genomic variation and its effect on phenotypes and gene regulation. *Nature* **477**, 289–294 (2011). 21921910 10.1038/nature10413 PMC3276836

[80] Davis, C. A. et al. The Encyclopedia of DNA Elements (ENCODE): data portal update. *Nucleic Acids Res.* **46**, D794–D801 (2018). 29126249 10.1093/nar/gkx1081 PMC5753278

[81] Strona, G., Nappo, D., Boccacci, F., Fattorini, S. & San-Miguel-Ayanz, J. A fast and unbiased procedure to randomize ecological binary matrices with fixed row and column totals. *Nat. Commun.* **5**, 4114 (2014). 24916345 10.1038/ncomms5114

[82] Hao, Y. et al. Integrated analysis of multimodal single-cell data. *Cell* **184**, 3573–3587.e29 (2021). 34062119 10.1016/j.cell.2021.04.048 PMC8238499

[83] Stuart, T., Srivastava, A., Madad, S., Lareau, C. A. & Satija, R. Single-cell chromatin state analysis with Signac. *Nat. Methods* **18**, 1333–1341 (2021). 34725479 10.1038/s41592-021-01282-5 PMC9255697

[84] Korsunsky, I. et al. Fast, sensitive and accurate integration of single-cell data with Harmony. *Nat. Methods* **16**, 1289–1296 (2019). 31740819 10.1038/s41592-019-0619-0 PMC6884693

[85] Fryzlewicz, P. Detecting possibly frequent change-points: wild binary segmentation 2 and steepest-drop model selection—rejoinder. *J. Korean Stat. Soc.* **49**, 1099–1105 (2020). 32952406 10.1007/s42952-020-00085-2 PMC7493064

