# Supplementary material for: Retrospective and multifactorial single-cell profiling reveals sequential chromatin reorganization during X inactivation
Source: Nat Cell Biol. 2025 Jul 10;27(7):1186–98. doi: 10.1038/s41556-025-01687-w (PMC12270907; doi:10.1038/s41556-025-01687-w)
Supplement: Supplementary file 1 — Reporting Summary [file 41556_2025_1687_MOESM1_ESM.pdf]

nature portfolio

Corresponding author(s): Peter Zeller

Last updated by author(s): Apr 14, 2025

Reporting Summary

Nature Portfolio wishes to improve the reproducibility of the work that we publish. This form provides structure for consistency and transparency in reporting. For further information on Nature Portfolio policies, see our Editorial Policies and the Editorial Policy Checklist.

Statistics

For all statistical analyses, confirm that the following items are present in the figure legend, table legend, main text, or Methods section.

n/a	Confirmed	
☐	☒	The exact sample size (*n*) for each experimental group/condition, given as a discrete number and unit of measurement
☒	☐	A statement on whether measurements were taken from distinct samples or whether the same sample was measured repeatedly
☐	☒	The statistical test(s) used AND whether they are one- or two-sided<br>*Only common tests should be described solely by name; describe more complex techniques in the Methods section.*
☒	☐	A description of all covariates tested
☐	☒	A description of any assumptions or corrections, such as tests of normality and adjustment for multiple comparisons
☐	☒	A full description of the statistical parameters including central tendency (e.g. means) or other basic estimates (e.g. regression coefficient) AND variation (e.g. standard deviation) or associated estimates of uncertainty (e.g. confidence intervals)
☐	☒	For null hypothesis testing, the test statistic (e.g. *F*, *t*, *r*) with confidence intervals, effect sizes, degrees of freedom and *P* value noted<br>*Give P values as exact values whenever suitable.*
☒	☐	For Bayesian analysis, information on the choice of priors and Markov chain Monte Carlo settings
☒	☐	For hierarchical and complex designs, identification of the appropriate level for tests and full reporting of outcomes
☐	☒	Estimates of effect sizes (e.g. Cohen's *d*, Pearson's *r*), indicating how they were calculated

Our web collection on statistics for biologists contains articles on many of the points above.

Software and code

Policy information about availability of computer code

Data collection

BD FACS™ Sortware 1.2.0.142 was used to collect data from the FACS machine during cell sorting.
Bowtie2 (2.4.1)
Hisat2 (2.1.0)
samtools (1.17)
scDamAndTools (1.0)
seqtk (1.3)

Data analysis	Python (3.8.2)<br>scDamAndTools (1.0)<br>NumPy (1.24.4)<br>Pandas (2.0.3)<br>Scipy (1.10.1)<br>h5py (3.11.0)<br>Scanpy (1.9.8)<br>Scvelo (0.3.2)<br>Matplotlib (3.7.5)<br>Seaborn (0.13.2)<br>Pygbrowse (0.3.3)<br>Glob (0.7)<br>Math (1.0.0)<br>Custom code for data processing and downstream analysis available at https://github.com/KindLab/DamChIC

For manuscripts utilizing custom algorithms or software that are central to the research but not yet described in published literature, software must be made available to editors and reviewers. We strongly encourage code deposition in a community repository (e.g. GitHub). See the Nature Portfolio guidelines for submitting code & software for further information.

Data

Policy information about availability of data

All manuscripts must include a data availability statement. This statement should provide the following information, where applicable:

- Accession codes, unique identifiers, or web links for publicly available datasets
- A description of any restrictions on data availability
- For clinical datasets or third party data, please ensure that the statement adheres to our policy

All data generated in this study has been uploaded to Gene Expresion Omnibus (GEO) accession number: GSE247458 and GSE288852

A number of previously published datasets were used in this study that were downloaded from the following public depositories:
- K562 H3K4me3 ChIP-seq: ENCSR668LDD
- K562 H3K27me3 ChIP-seq: ENCSR000EWB
- K562 H3K9me3 ChIP-seq: ENCSR000APE
- K562 ATAC-seq: ENCSR956DNB
- multi-CUT&Tag: GSE171554
- MulTI-Tag: GSE179756
- NTT-seq: GSE212588
- Nano-CT: GSE198467
- CHART-seq: GSE48649

For human experiments reference genome hg19 (GRCh37) was used and for mouse mm10 (GRCm38).
Alu/SINE and L1/LINE annotations were obtained from the RepeatMasker database (repeatmasker.org)

Human research participants

Policy information about studies involving human research participants and Sex and Gender in Research.

Reporting on sex and gender	No human research participants
Population characteristics	NA
Recruitment	NA
Ethics oversight	NA

Note that full information on the approval of the study protocol must also be provided in the manuscript.

Field-specific reporting

Please select the one below that is the best fit for your research. If you are not sure, read the appropriate sections before making your selection.

☒ Life sciences ☐ Behavioural & social sciences ☐ Ecological, evolutionary & environmental sciences

For a reference copy of the document with all sections, see nature.com/documents/nr-reporting-summary-flat.pdf

Life sciences study design

All studies must disclose on these points even when the disclosure is negative.

Sample size	Sample size was estimated to result in at least 50 cells per cell cluster and condition.
Data exclusions	Cells that did not pass quality controls were excluded from the analysis. To exclude single-cell samples from the analysis that failed we applied construct-specific cutoffs on UMI-unique reads for scDamID: 1,000 for Dam and Dam-scFv-H3K27me3 (Rang et al., 2022), and 5,000 for Dam-LMNB1. A general cutoff of 1,000 UMI-unique reads per sample was applied for ChIC. Samples in allele-specific analysis were included if ≤200 UMI-unique reads could be assigned to both parental alleles for scDamID as well as sortChIC-derived reads.
Replication	We performed experiments across multiple plates and found the results across these minimal three technical replicates to be reproducible. When projecting cells across technical replicates onto a low-dimensional manifold, we did not observe effects coming from differences in technical replicates.
Randomization	We minimized effects across plates by sorting different different time points of time course expeirments onto the same plate. The location of each cell was not randomly assigned to the well on the plate.
Blinding	No blinding was used in this study. Obtained data was compared in detail to publicly available dataset to validate the result. All data analysis was done automated.

Reporting for specific materials, systems and methods

We require information from authors about some types of materials, experimental systems and methods used in many studies. Here, indicate whether each material, system or method listed is relevant to your study. If you are not sure if a list item applies to your research, read the appropriate section before selecting a response.

Materials & experimental systems

n/a	Involved in the study	
☐	☒	Antibodies
☐	☒	Eukaryotic cell lines
☒	☐	Palaeontology and archaeology
☒	☐	Animals and other organisms
☒	☐	Clinical data
☒	☐	Dual use research of concern

Methods

n/a	Involved in the study	
☐	☒	ChIP-seq
☐	☒	Flow cytometry
☒	☐	MRI-based neuroimaging

Antibodies

Antibodies used	Rabbit polyclonal anti-Lamin B1 (Abcam, ab16048), used 1:200 or 1:400<br>Rabbit polyclonal anti-H3K4me1 (Abcam, ab8895), used 1:400<br>Rabbit monoclonal anti-H3K27me3 (Cell Signaling Technologies, 9733S), used 1:200<br>Rabbit monoclonal anti-H3K9me3 RM389 (Thermofisher, MA5-33395), used 1:200<br>Rabbit monoclonal anti-H3K4me3 (Thermofisher, MA5-11199), used 1:400<br>Rabbit monoclonal anti-Histone H3 (Abcam, ab176842), used 1:400<br>Rabbit monoclonal anti-H2AK119Ub (Cell Signaling, D27C4)
Validation	We validated antibodies by performing Dam&ChIC on Kbm7 cells and confirmed that we reproduced the publicly available ChIP-seq signals from the ENCODE project.

Eukaryotic cell lines

Policy information about cell lines and Sex and Gender in Research

Cell line source(s)	Human haploid KBM7 cells (Kotecki et al. 1999)<br>Human haploid KBM7 cells expressing DD-Dam-LMNB1 (Kind et al., 2015)<br>Human haploid KBM7 cells expressing DD-Dam (Kind et al., 2015)<br>Mouse F1 hybrid Cast/EiJ x 129/Sv ESCs expressing AID-Dam-LMNB1; Tir1 knock-in in Tigre locus, endogenous knock-in of Dam in the LMNB1 locus (Guerreiro et al., 2023)<br>Mouse F1 hybrid Cast/EiJ x 129/Sv ESCs expressing AID-Dam-scFv-K27me3-ER: Tir1 knock-in in Tigre locus, scFv-H3K27me3-ER knock-in Rosa26 locus (Rang et al., 2022)
Authentication	The original KBM7 cell line was authenticated by Karyotyping and genome content determination by PI staining over multiple generations. KBM7 transgenic cell lines were authenticated using DpnII-qPCR for transgene function and PI staining for haploid genome content. Mouse F1 hybrid Cast/EiJ x 129/Sv ESCs expressing AID-Dam-LMNB1 and mouse F1 hybrid Cast/EiJ x

	129/Sv ESCs expressing AID-Dam-scFv-K27me3-ER were authenticated previously by karyo-seq and by PCR and sequencing for correct transgene integration.
Mycoplasma contamination	Mycoplasma contamination was regular tested for and never detected in the cell lines used.
Commonly misidentified lines (See ICLAC register)	no commonly misidentified lines were used.

ChIP-seq

Data deposition

☒ Confirm that both raw and final processed data have been deposited in a public database such as GEO.

☒ Confirm that you have deposited or provided access to graph files (e.g. BED files) for the called peaks.

Data access links
May remain private before publication.

https://www.ncbi.nlm.nih.gov/geo/query/acc.cgi?acc=GSE247458

Files in database submission

GSM7902805	KBM7 Dam&ChIC Dam-LMNB1/H3K9me3 (HMF1477.index12)
GSM7902806	KBM7 Dam&ChIC Dam/H3K9me3 (HMF1477.index14)
GSM7902807	KBM7 Dam&ChIC Dam-LMNB1/H3K27me3 (KIN5917.index31)
GSM7902808	KBM7 Dam&ChIC Dam-LMNB1/H3K27me3 (KIN5917.index43)
GSM7902809	KBM7 Dam&ChIC Dam-LMNB1/LMNB1 (KIN6062.index10)
GSM7902810	KBM7 sortChIC LMNB1 (KIN6062.index11)
GSM7902811	KBM7 scDamID Dam-LMNB1 (KIN6062.index13)
GSM7902812	KBM7 Dam&ChIC Dam-LMNB1/LMNB1 (KIN6062.index19)
GSM7902813	KBM7 Dam&ChIC Dam-LMNB1/LMNB1 (KIN6062.index20)
GSM7902814	KBM7 Dam&ChIC Dam-LMNB1/LMNB1 (KIN6062.index21)
GSM7902815	KBM7 Dam&ChIC Dam-LMNB1/H3K4me3 (KIN6214.index30)
GSM7902816	KBM7 Dam&ChIC Dam-LMNB1/H3K9me3 (KIN6214.index34)
GSM7902817	KBM7 Dam&ChIC Dam-LMNB1/H3K9me3 (KIN6668.index19)
GSM7902818	KBM7 Dam&ChIC Dam-LMNB1/H3K4me3 (KIN6668.index22)
GSM7902819	KBM7 Dam&ChIC Dam-LMNB1/H3K4me3 (KIN6668.index23)
GSM7902820	KBM7 Dam&ChIC Dam-LMNB1/H3K9me3 (KIN7781.index46)
GSM7902821	KBM7 mitosis Dam&ChIC Dam-LMNB1/LMNB1
GSM7902822	KBM7 mitosis Dam&ChIC Dam-LMNB1/LMNB1
GSM7902823	KBM7 mitosis Dam&ChIC Dam-LMNB1/LMNB1
GSM7902824	KBM7 mitosis Dam&ChIC Dam-LMNB1/LMNB1
GSM7902825	KBM7 mitosis Dam&ChIC Dam-LMNB1/LMNB1
GSM7902826	KBM7 Dam&ChIC Dam/H3K27me3 (KIN8791_kbm7.index42)
GSM7902827	KBM7 Dam&ChIC Dam/H3K27me3 (KIN8791_kbm7.index43)
GSM7902828	KBM7 Dam&ChIC Dam/H3K4me3 (KIN8791_kbm7.index44)
GSM7902829	KBM7 Dam&ChIC Dam/H3K4me3 (KIN8791_kbm7.index45)
GSM7902830	KBM7 Dam&ChIC Dam/H3K9me3 (KIN8791_kbm7.index46)
GSM7902831	VitC Dam&ChIC Dam-LMNB1/H3K27me3 (KIN8164.index15)
GSM7902832	VitC Dam&ChIC Dam-LMNB1/H3K27me3 (KIN8164.index16)
GSM7902833	VitC Dam&ChIC Dam-LMNB1/LMNB1 (KIN8403.index35)
GSM7902834	VitC Dam&ChIC Dam-LMNB1/LMNB1 (KIN8403.index36)
GSM7902835	VitC Dam&ChIC Dam-scFv-H3K27me3/H3K9me3 (KIN8403.index45)
GSM7902836	VitC Dam&ChIC Dam-scFv-H3K27me3/H3K9me3 (KIN8403.index46)
GSM7902837	VitC Dam&ChIC Dam-LMNB1/H3K27me3 (KIN8433_xci.index16)
GSM7902838	VitC Dam&ChIC Dam-LMNB1/H3K27me3 (KIN8433_xci.index18)
GSM7902839	VitC Dam&ChIC Dam-LMNB1/H3K27me3 (KIN8433_xci.index19)
GSM7902840	VitC Dam&ChIC Dam-LMNB1/H3K27me3 (KIN8433_xci.index20)
GSM7902841	VitC Dam&ChIC Dam-scFv-H3K27me3/LMNB1 (KIN8791_KIN8928_xci.index21)
GSM7902842	VitC Dam&ChIC Dam-scFv-H3K27me3/LMNB1 (KIN8791_KIN8928_xci.index22)
GSM7902843	VitC Dam&ChIC Dam-scFv-H3K27me3/LMNB1 (KIN8791_KIN8928_xci.index23)
GSM7902844	VitC Dam&ChIC Dam-scFv-H3K27me3/LMNB1 (KIN8791_KIN8928_xci.index27)
GSM7902845	VitC Dam&ChIC Dam-scFv-H3K27me3/LMNB1 (KIN8791_KIN8928_xci.index28)
GSM7902846	VitC Dam&ChIC Dam-scFv-H3K27me3/LMNB1 (KIN8791_KIN8928_xci.index29)
GSM7902847	VitC Dam&ChIC Dam-LMNB1/LMNB1 (KIN8791_KIN8928_xci.index30)
GSM7902848	VitC Dam&ChIC Dam-LMNB1/LMNB1 (KIN8791_KIN8928_xci.index31)
GSM7902849	VitC Dam&ChIC Dam-LMNB1/LMNB1 (KIN8791_KIN8928_xci.index32)
GSM7902850	VitC Dam&ChIC Dam-LMNB1/LMNB1 (KIN8791_KIN8928_xci.index33)
GSM7902851	VitC Dam&ChIC Dam-LMNB1/LMNB1 (KIN8791_KIN8928_xci.index34)
GSM7902852	VitC Dam&ChIC Dam-LMNB1/LMNB1 (KIN8791_KIN8928_xci.index35)
GSM7902853	VitC Dam&ChIC Dam-LMNB1/H3K9me3 (KIN8791_KIN8928_xci.index36)
GSM7902854	VitC Dam&ChIC Dam-LMNB1/H3K9me3 (KIN8791_KIN8928_xci.index37)
GSM8776804	VitC Dam&ChIC Dam-LMNB1/H2AK119Ub (KIN9826.index44)
GSM8776805	VitC Dam&ChIC Dam-LMNB1/H2AK119Ub (KIN9826.index45)
GSM8776806	VitC Dam&ChIC Dam-LMNB1/H2AK119Ub (KIN10162_xci.index47)
GSM8776807	VitC Dam&ChIC Dam-LMNB1/H2AK119Ub (KIN10162_xci.index48)
GSM8776808	VitC scDam&T-seq Dam-LMNB1 (VAN10206.index27)
GSM8776809	VitC scDam&T-seq Dam-LMNB1 (VAN10206.index28)

	GSM8776810 VitC scDam&T-seq Dam-LMNB1 (VAN10206.index30)<br>GSM8776811 VitC scDam&T-seq Dam-LMNB1 (VAN10206.index32)<br>GSM8776812 VitC scDam&T-seq Dam-LMNB1 (VAN10206.index34)<br>GSM8776813 VitC scDam&T-seq Dam-LMNB1 (VAN10206.index36)
Genome browser session (e.g. UCSC)	NA

Methodology

Replicates	All experiments were performed over at least 3 technical replicates (plates).
Sequencing depth	Plates were sequenced to a depth of 30-40M raw reads.
Antibodies	Rabbit polyclonal anti-Lamin B1 (Abcam, ab16048)<br>Rabbit polyclonal anti-H3K4me1 (Abcam, ab8895)<br>Rabbit monoclonal anti-H3K27me3 (Cell Signaling Technologies, 9733S)<br>Rabbit monoclonal anti-H3K9me3 RM389 (Thermofisher, MA5-33395)<br>Rabbit monoclonal anti-H3K4me3 (Thermofisher, MA5-11199)<br>Rabbit monoclonal anti-Histone H3 (Abcam, ab176842)<br>Rabbit monoclonal anti-H2AK119Ub (Cell Signaling, D27C4)
Peak calling parameters	For peak calling Macs2 was used in `bdgpeakcall` mode with default parameters.
Data quality	Cells that did not pass quality controls were excluded from the analysis. To exclude single-cell samples from the analysis that failed we applied construct-specific cutoffs on UMI-unique reads for scDamID: 1,000 for Dam and Dam-scFv-H3K27me3 (Rang et al., 2022), and 5,000 for Dam-LMNB1. A general cutoff of 1,000 UMI-unique reads per sample was applied for ChIC. Samples in allele-specific analysis were included if ≤200 UMI-unique reads could be assigned to both parental alleles for scDamID as well as sortChIC-derived reads.
Software	packages and code for analysis: data processing steps and downstream analysis available at https://github.com/KindLab/DamChIC

Flow Cytometry

Plots

Confirm that:

☒ The axis labels state the marker and fluorochrome used (e.g. CD4-FITC).

☒ The axis scales are clearly visible. Include numbers along axes only for bottom left plot of group (a 'group' is an analysis of identical markers).

☒ All plots are contour plots with outliers or pseudocolor plots.

☒ A numerical value for number of cells or percentage (with statistics) is provided.

Methodology

Sample preparation	Ethanol fixed where stained with combination of CellTrace far-red, yellow and CFSE before pooling. Fixed cells or isolated nuclei were spun at 400 g for 5 minutes and washed once with 400 microlitre Wash Buffer 1 (47.5 ml H2O RNAse free, 1 ml 1 M HEPES pH 7.5 (Invitrogen), 1.5 ml 5M NaCl, 3:6 µl pure spermidine solution (Sigma Aldrich), 0.05% saponin (nuclei) or 0.05% Tween20 (fixed cells), 2mM EDTA). Cells were spun again at 400 g and resuspended in 400 microlitre Wash Buffer 1. Cell suspension was incubated with indicated chromatin antibody overnight on a roller at 4 degrees Celsius. The next day cells were spun at 400 g, washed once with 400 microlitre Wash Buffer 2 (Wash Buffer 1 without EDTA) and resuspended in 500 microlitre Wash Buffer 2 containing pA-MNase (3 ng/mL) and Hoechst 34580 (2.5 ug/ml) and incubated for 1 hour on a rotator at 4 degrees Celsius.<br><br>Finally, samples were washed twice with 500 microlitre Wash Buffer 2 before passing them through a 70 micron cell strainer (Corning, 431751) and sorting on a BD Influx FACS machine, with CellTrace and cell cycle specific gating, into 384 well plates containing 100 nanoliter Wash buffer 3 (Wash buffer containing 0.05 % Saponin (nuclei) or Tween (fixed cells)) and 5 microlitre sterile filtered mineral oil (Sigma Aldrich) per well. Plates where spun for 1min with 2000g at 4C.
Instrument	BD Influx System
Software	BD FACS™ Sortware 1.2.0.142
Cell population abundance	In case of pooled processing of multiple conditions, samples where pooled at equal ratios, which was confirmed during FACS sorting.
Gating strategy	We used a forward scatter gate to remove debris (low FSC) and trigger pulse width to remove doublets (high trigger pulse width). We selected cell based on Hoechst staining on their cell cycle stage and used the combination of Cell-tracer far red, yellow and CFSE to distinguish experimental conditions.

☒ Tick this box to confirm that a figure exemplifying the gating strategy is provided in the Supplementary Information.
